# 24-hour-restraint stress induces long-term depressive-like phenotypes in mice

**DOI:** 10.1038/srep32935

**Published:** 2016-09-09

**Authors:** Xixia Chu, Ying Zhou, Zhiqiang Hu, Jingyu Lou, Wei Song, Jing Li, Xiao Liang, Chen Chen, Shuai Wang, Beimeng Yang, Lei Chen, Xu Zhang, Jinjing Song, Yujie Dong, Shiqing Chen, Lin He, Qingguo Xie, Xiaoping Chen, Weidong Li

**Affiliations:** 1 Bio-X Institutes, Key Laboratory for the Genetics of Development and Neuropsychiatric Disorders (Ministry of Education), Shanghai Key Laboratory of Psychotic Disorders, and Brain Science and Technology Research Center, Shanghai Jiao Tong University, 800 Dongchuan Road, Shanghai 200240, China; 2 School of Life Science and Biotechnology, Shanghai Jiao Tong University, 800 Dongchuan Road, Shanghai 200240, China; 3 Huazhong University of Science and Technology, 1037 Luoyu Road, Wuhan 430074, Hubei, China; 4 National Key Laboratory of Human Factors Engineering, China Astronaut Research and Training Center, Beijing 100094, China; 5 Wuxi Mental Health Center, 156 Qianrong Road, Wuxi 214151, Jiangsu, China

## Abstract

There is an increasing risk of mental disorders, such as acute stress disorder (ASD), post-traumatic stress disorder (PTSD) and depression among survivors who were trapped in rubble during earthquake. Such long-term impaction of a single acute restraint stress has not been extensively explored. In this study, we subjected mice to 24-hour-restraint to simulate the trapping episode, and investigated the acute (2 days after the restraint) and long-term (35 days after the restraint) impacts. Surprisingly, we found that the mice displayed depression-like behaviors, decreased glucose uptake in brain and reduced adult hippocampal neurogenesis 35 days after the restraint. Differential expression profiling based on microarrays suggested that genes and pathways related to depression and other mental disorders were differentially expressed in both PFC and hippocampus. Furthermore, the depression-like phenotypes induced by 24-hour-restraint could be reversed by fluoxetine, a type of antidepressant drug. These findings demonstrated that a single severe stressful event could produce long-term depressive-like phenotypes. Moreover, the 24-hour-restraint stress mice could also be used for further studies on mood disorders.

Natural disasters, such as earthquakes, not only lead to great devastation and enormous deaths and injuries, but also pose psychological consequences to people^1^. Survivors from the disaster are more likely to develop stress-related disorders, including ASD, PTSD, substance abuse and depression^2^,^3^,^4^,^5^,^6^. Over the two decades, studies about depression after a disaster have demonstrated that the prevalence of depression range from 4.9% to 54%, persisting for weeks, months or even years^7^. Functional magnetic resonance imaging (fMRI) studies showed the connectivity among prefrontal-limbic systems was increased but limbic-striatal areas and default-mode areas were attenuated in earthquake survivors^8^. Another study found the white matter microstructure in right prefrontal lobe and parahippocampus was decreased after less than a month of the earthquake^9^. These reports showed that traumatic experiences could affect the fronto-limbic network involved in emotion regulation. However, most researches about mental health of earthquake survivors largely relied on surveys and neuroimaging studies, which could not reveal the specific biological mechanism of how traumatic experience about earthquake leads to mood disorders.

Various risk factors of mood disorders (like PTSD or depression) in survivors after the earthquake have been investigated, and some studies have demonstrated that being trapped in the earthquake is a strong or even the strongest risk factor of mental disorders^1^,^3^,^7^,^10^. Based on these results, we decided to subject mice to 24 hours restraint to mimic the situation victims being trapped under the rubble. Restraint is commonly used to stress animals^11^. Previous studies have revealed that body temperature, arterial pressure, heart rate and serum corticosterone levels were increased during/after acute restraint stress^12^,^13^. In contrast, aggressive conflicts, motor activity and exploration were generally found decreased among stressed animals^13^,^14^. Acute stress could also induce dendritic remodeling and reduce phosphorylated cofilin in medial prefrontal cortex^15^. Moreover, alternations of immune, neurogenesis, cognition and memory in restraint animals also indicated multiple influences of acute stress on immunity system and neuronal plasticity^16^,^17^,^18^. However, the long-term impact of a single severe restraint stress has not been extensively explored.

In this study, we subjected mice to 24 hours restraint to simulate the episode survivors trapped under collapsed buildings after an earthquake. Both behavioral and molecular changes were investigated to reveal the acute (2 days after the restraint) and long-term (5 weeks after the restraint) impact of the 24-hour-restraint. Interestingly, we found that the 24-hour-restraint stress could produce long-term depressive-like phenotypes, which could be reversed by fluoxetine, a type of antidepressant drug. The 24-hour-restraint stress mice could also be used as a feasible mouse model for further studies on mood disorders, like PTSD or depression.

## Result

### Depressive-like behaviors induced by 24-hour restraint

Mice were subjected to 24 hours restraint to simulate the episode victims being trapped. During the restraint, we ensured that the mice’s bodies and hindquarters could not move or turn around, but their head and anterior limb could move. And the mice had no access to food and water. The details of restraint procedure are described in Methods and the apparatus and illustrations of the restraint are shown in Supplementary Figure S1.

To study the impact of the restraint, we measured the corticosterone (CORT) level in serum at different time points during the 24-hour-restraint. As a result, the CORT was immediately elevated at 1 hour after the beginning of the restraint, and reached to ~2 fold at the end of the restraint (Supplementary Figure S2). After 24 hours’ recovery, the CORT level went back to the normal level. Previous studies also showed that acute stress would activate the hypothalamus-pituitary-adrenal (HPA) axis, producing a transient increase of CORT in rodent^19^. Besides, significant body weight loss (26.7%) was found in the restraint mice immediately after the 24-hour-restraint (Supplementary Figure S3), largely as a result of the deprivation of food and water. The body weight returned to the normal level after one day’s recovery. However, it showed a slight (3.4%) but significant weight gain 35 days after the restraint (Supplementary Figure S3). These results suggested the 24-hour-restraint is a strong acute stress.

**Figure 1:**
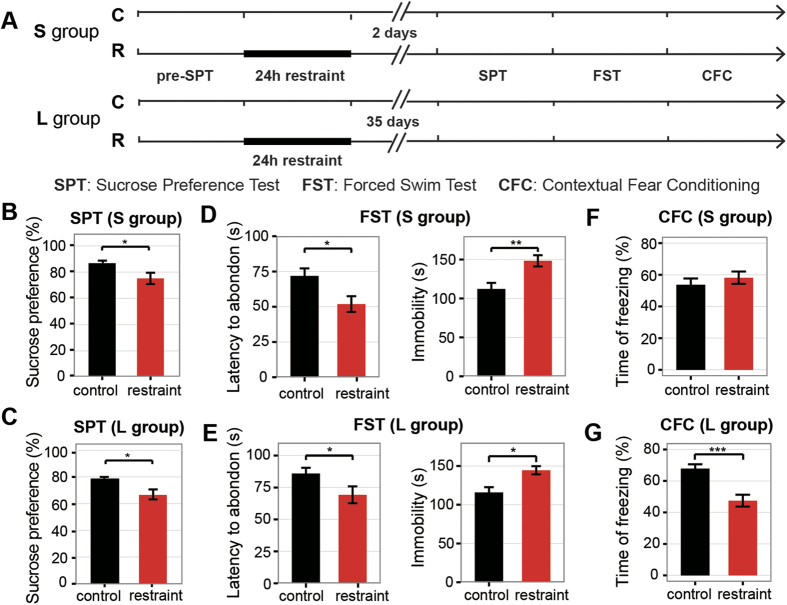
The 24-hour-restraint stress induced depressive-like behaviors. (**A**) Experiment designs to test the short-term (S group) and long-term (L group) impact of the restraint on mice. (**B–C**) Sucrose preference percentage of 24 hours in sucrose preference test of S group (**B**) and L group (**C**). The p-value (t-test) for S group is <0.05 (N = 24 vs. 25) and L group is <0.05 (N = 23 vs. 25). (**D–E**) The struggling time before the first abandon and total time of immobility in the forced swimming test of S group (**D**) and L group (**E**). The p-values of t-test of first abandon are <0.05 (N = 24 vs. N = 25) and <0.01(N = 25 vs. 28) respectively. And the p-values of t-test of immobility are <0.01 (N = 24 vs. N = 25) and <0.01 (N = 26 vs. 28) respectively. (**F**,**G)** The percentage of freezing time in the contextual fear conditioning test for the S group (**F**) and L group (**G**). The p-values of t-test are 0.30 (N = 21 vs. 23) and <0.001 (N = 20 vs. 21) respectively. Data are presented as mean ± SEM, *P < 0.05, **P < 0.01, ***P < 0.001.

In order to distinguish the short-term and long-term effects of the restraint on animals’ behaviors, we separated mice into two groups (S group and L group) (Fig. 1A). Behavioral tests on S group were carried out at 2 days after the restraint, while behavioral tests on L group were performed at 35 days after the restraint. Before the 24-hour-restraint, we conducted sucrose preference test to measure the level of experiencing pleasure. As a result, there’s no difference of sucrose preference between the unrestraint mice and the control before the 24-hour-restraint (Supplementary Figures S4 and S5). However, after the restraint, mice displayed a decreased sucrose preference than the controls in both S group and L group (Fig. 1B,C). These results suggested the restraint mice lack reactivity to pleasure, known as anhedonia, which is a core symptom of depression^20^. In the forced swimming test, a measure of behavioral despair, reduced latency to abandon and increased immobility were found in the restraint mice for both S group (Fig. 1D) and L group (Fig. 1E). Moreover, we carried out the contextual fear-conditioning (CFC) test to assess the fear memory. There was no difference in freezing time between the restraint and the control mice in S group (Fig. 1F). Interestingly, the restraint mice exhibited less freezing time in L group (Fig. 1G). Previous study also reported that animals exposed to chronic stress showed deficits in learning and cognition flexibility^21^. The CFC tests indicated that the 24-hour restraint could induce abnormal cognition function and the visible abnormality occurred after long-time accumulation.

Furthermore, in other mood- and cognition- related behavioral tests, including open field test (OFT), elevated plus maze (EPM), social interaction test (SIT) and prepulse inhibition (PPI) test, we found no significant difference between restraint and control animals in both S group and L group (Supplementary Figure S6 and S7).

These findings implied that the 24-hour-restraint could induce depressive-like phenotypes, and the effects of the stress on behaviors were long lasting. More importantly, some defects (like cognitive memory) may be only visible after long-time accumulation of abnormal biological changes.

### Alternation of glucose uptake of brain revealed by PET

**Figure 2:**
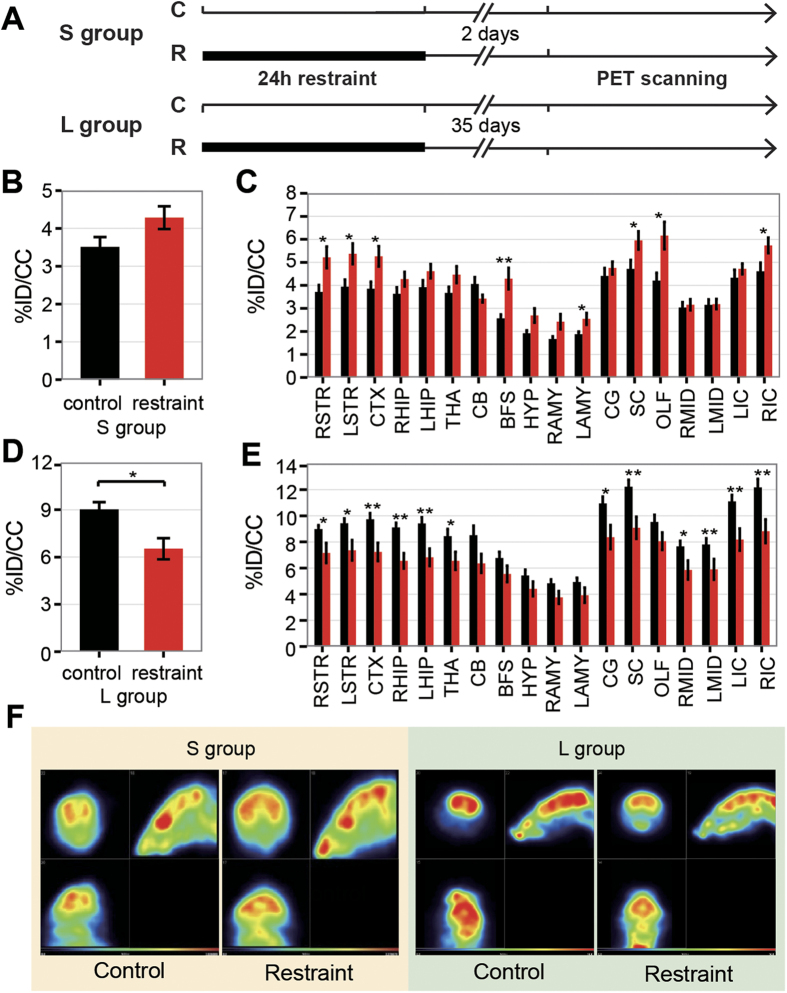
Alternations of glucose metabolism by small animal PET scanning. Glucose uptake is measured as %ID/cc (%injected dose per mL of tissue). (**A**) Experiment design of the PET scanning. (**B**) The average glucose uptake of the whole brain of S group shows no difference between control and restraint mice (N = 7 vs. 9). (**C**) The changes of glucose uptake in brain regions of S group. Glucose uptake of brain regions including STR, CTX, BFS, LAMY, SC, OLF, RIC is significantly increased in restraint mice. (**D**) The average glucose uptake of the whole brain for L group is significantly decreased in restraint mice (N = 8 vs. N = 7), p value of student t-test is<0.01. (**E**) The glucose uptake of different brain regions of L group. Brain regions such as STR, CTX, HIP, THA, CG, SC, MID and IC were significantly decreased in restraint mice. (**F**) Representatives of the glucose uptake of S and L group. Data are presented as mean ± SEM, *P < 0.05, **P < 0.01. Abbreviations: RSTR, right striatum; LSTR, left striatum; CTX, cortex; RHIP, right hippocampal region; LHIP, left hippocampal region; THA, thalamus; CB, cerebellum; BFS, basal forebrain/septum; HYP, hypothalamus; RAMY, right amygdala; LAMY, left amygdala; CG, cingulate gyrus; SC, superior colliculus; OLF, olfactory areas; RMID, right midbrain; LMID, left midbrain; LIC, left inferior colliculus; RIC, right inferior colliculus.

**Figure 3:**
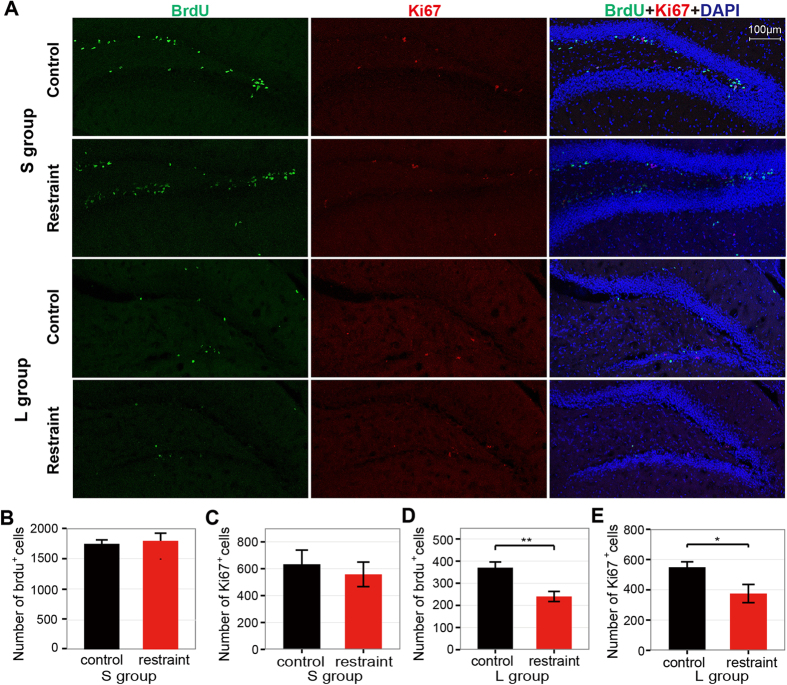
Effects of the 24-hour-restraint stress on adult neurogenesis. (**A**) Representative staining of BrdU (green), Ki67 (red) and DAPI (blue) in DG of hippocampus. (**B–C**) The numbers of BrdU^+^ (**B**) and Ki67^+^ (**C**) cells of S group display no difference between control and restraint mice. The p-values are 0.76 (N = 6 vs. 7) and 0.61 (N = 4 vs. 4) respectively. (**D–E**) The numbers of BrdU^+^ (**D**) and Ki67^+^ (**E**) cells of L group are decreased in the restraint mice. The p-values are <0.01 (N = 7 vs. 7) and <0.05 (N = 4 vs. 4) respectively. Data are presented as mean ± SEM, *P < 0.05, **P < 0.01.

Neurons account for the majority energy consumption of brain, therefore the dynamic changes of energy demand reflex the alteration in neuronal activation, which can be measured by^18^ F-fuoro-2-deoxyglucose (FDG)-PET scanning^22^. Hence we checked the brain glucose metabolism by small-animal PET scanning (Fig. 2A). In S group, the glucose uptake of specific brain regions were increased in restraint mice, including striatum (STR), cortex (CTX), basal forebrain/septum (BS), left amygdala (LAMY), superior colliculus (SC), olfactory areas (OLF) and right inferior colliculus (RIC) (Fig. 2C). However the average glucose uptake of the whole brain showed no significant difference (Fig. 3B). While in L group, it was significantly decreased in the restraint mice (Fig. 2D). The brain regions with decreased glucose uptake consist of STR, CTX, SC and IC, which were increased in the S group. In addition, brain regions including hippocampal region (HIP), thalamus (THA), cingulate gyrus (CG) and midbrain (MID) were also found to display decreased glucose uptake in L group (Fig. 2E). Our results showed that 24-hour-restraint could alter the glucose uptake of brain, differently in short term and long term.

### Decreased adult neurogenesis in long-term dentate gyrus

In the last decade, there are increasing evidences demonstrating the important role of adult hippocampal neurogenesis in the pathogenesis and therapeutics of depression^23^,^24^. Since our results above demonstrated that the 24-hour-restraint mice exhibited depressive-like behaviors, we examined whether the 24-hour-restraint stress had an influence on adult hippocampal neurogenesis. Both BrdU and Ki67 were employed to label the newborn neurons (Fig. 3A), and BrdU was injected 3 times during the 24-hour restraint. In S group, there was no significant difference of BrdU-positive cells in dentate gyrus (DG) between the restraint and the control mice (Fig. 3B) as well as Ki67-positve cells (Fig. 3C). However, in L group, we found significant reduction of cell survival by BrdU labeling (Fig. 3D) and decreased cell proliferation by Ki67 labeling (Fig. 3E). These results demonstrated that the impact of 24-hour-restaint on adult hippocampal neurogenesis was long lasting.

### Differential expression profiling of the restraint mice

Neuroimaging studies of major depression on humans and animal models have identified abnormalities in prefrontal cortex (PFC) and hippocampus^25^,^26^,^27^. Decreased volume of hippocampus and PFC has been reported among patients suffering depression^26^,^27^. These two brain regions have been intensively studied with regard to stress, depression and antidepressant actions^28^.

**Figure 4:**
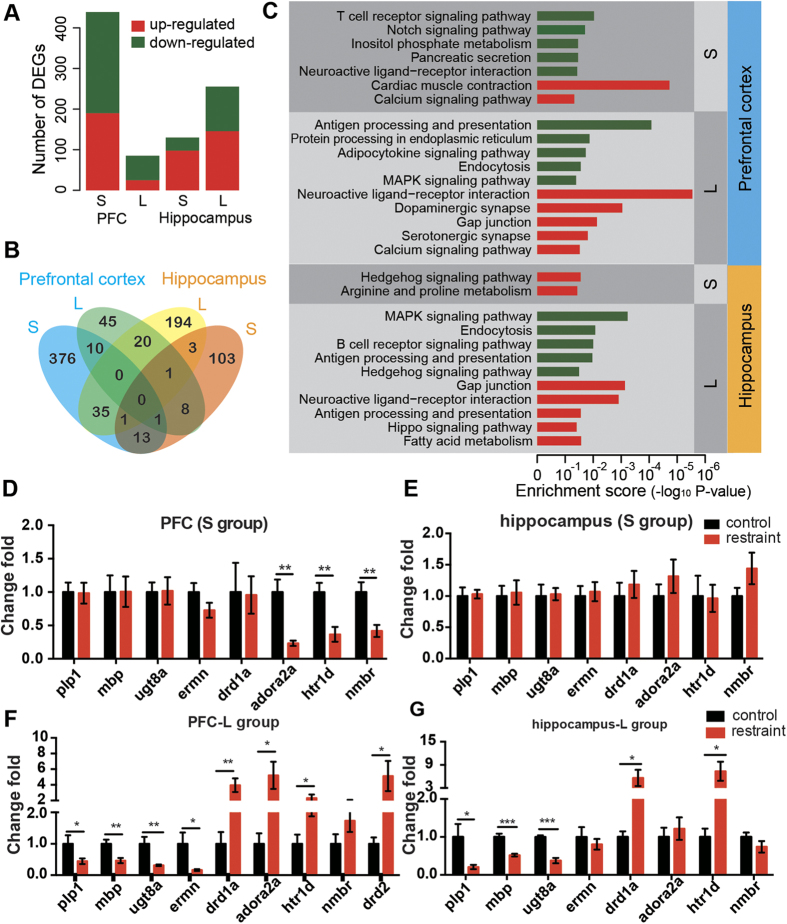
High-throughput gene expression analysis in short-term (S) and long-term (L) brain tissues (PFC and hippocampus) by microarrays. (**A**) Number of detected differentially expressed genes (DEGs). (**B**) Number of overlapping DEGs among short-term PFC, long-term PFC, short-term hippocampus and long-term hippocampus. (**C**) Enriched KEGG pathways revealed by the DEGs. Enrichments from up-regulated DEGs and down-regulated DEGs are shown in “red” and “green” respectively. (**D–E**). Fold changes of selected genes in PFC (**D**) and hippocampus (**E**) of S group measured by quantitative RT-PCR. F-G. Fold changes of selected genes in PFC (**F**) and hippocampus (**G**) of L group. Data are presented as mean ± SEM, *P < 0.05, **P < 0.01, ***P < 0.001.

In order to reveal the underlying molecular mechanism, we measured the gene profiles of the hippocampus and PFC of mice in S group and L group (N = 3 in each condition and N = 3*4 = 12 totally) by expression microarrays. We carried out differential expression analysis among each group. As a result, in the PFC, we detected 439 differentially expressed genes (DEGs, p-value < 0.05 and fold change>2) for S group experiment and 85 DEGs for L group experiment (Fig. 4A), indicating the effect of the restraint on the PFC declined after 5 weeks. In contrast, in the hippocampus, we detected 130 DEGs for S group experiment but more (255) DEGs in L group experiment (Fig. 4A). This suggested gene expressions of the hippocampus undergo dramatic changes after 5 weeks of the restraint. Moreover, few overlapped DEGs were found between S group experiments and L group experiments of the same tissues (Fig. 4B), further demonstrating the long-term changes after the restraint differed from short-term changes.

In addition, enrichment analysis based on KEGG pathways^29^ and Gene Ontology (GO)^30^ annotations were carried out (Fig. 4C, Supplementary Table S1 and S2). Interestingly, neuroactive ligand-receptor interaction pathway was enriched in down-regulated DEGs in PFC of S group. And it was also enriched in up-regulated DEGs in both PFC and hippocampus of L group. Besides, MAPK signaling pathway, endocytosis, antigen processing and presentation pathway were down-regulated and gap junction was up-regulated in both PFC and hippocampus of L group. We carried out quantitative real time PCR (qPCR) to verify expression changes of genes involved in neuroactive ligand-receptor interaction pathway (including *Drd1a*, *Drd2*, *Adora2a* and *Htr1d*, *Nmbr*). As a result, *Drd1a*, *Adora2a* and *Htr1d* gene expressions were significantly increased in PFC of L groups (Fig. 4F) but were decreased in PFC of S group (Fig. 4D). *Drd2* and *Nmbr* gene expressions were also up-regulated in PFC of L group but had no changes in S group (Fig. 4D). In addition, in the long-term hippocampus, *Drd1a* and *Htr1d* gene expressions were increased (Fig. 4G) but no difference were found in the short-term hippocampus (Fig. 4E). As is known, the dopamine D1 receptor^31^, D2 receptor^32^, the adenosine A2A receptor^31^ and serotonin receptors^33^,^34^ have long been associated with anxiety disorders.

Moreover, GO enrichment suggested decreased myelination (including *Plp1*, *Mbp*, *Ugt8a* and *Ermn*) of PFC and hippocampus in L group (Table S2), which were also verified by qPCR experiments (Fig. 4F,G). But there were no significant changes in S group (Fig. 4D,E). These signaling pathways and differentially expressed genes might underline the possible molecular mechanisms involved in the depressive-like phenotypes induced by the 24-hour-restraint and need further exploration.

### Reversal of depressive-like phenotypes by fluoxetine treatment

**Figure 5:**
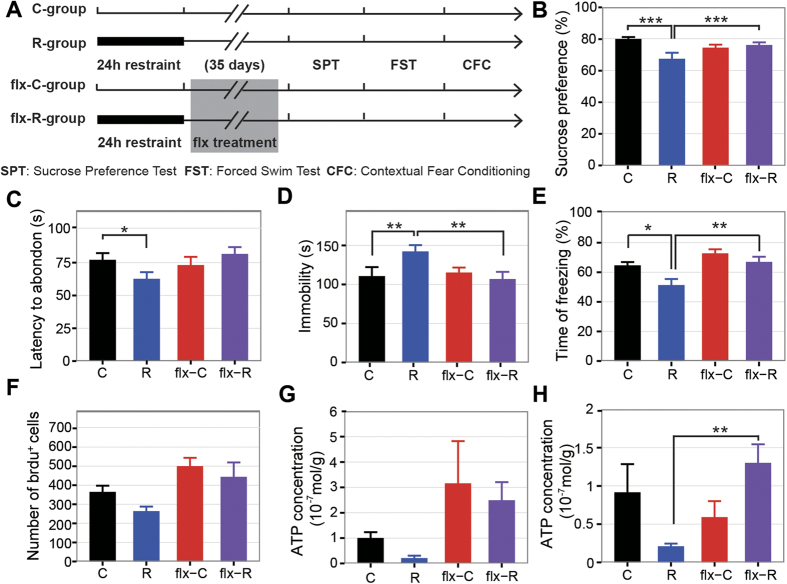
Fluoxetine treatment reverses the depressive-like phenotypes induced by 24-hour-restraint. (**A**) Experiment design in order to test the effect of fluoxetine (flx) treatment. (**B**) The sucrose consumption in the sucrose preference test. (2-way ANOVA, stress × durg: p < 0.01, drug: p = 0.03; stress: p < 0.01, N = C:23, R:28, flx-C:28, flx-R:28). (**C**) The struggling time before the first abandon in the forced swim test (2-way ANOVA, stress×durg: p = 0.2271; drug: p = 0.092; stress: p < 0.01; N = C:30, R:30, flx-C:29, flx-R:30). (**D**) The total time of immobility in the forced swim test (2-way ANOVA, stress×durg: p < 0.01; drug: p = 0.095; stress: p < 0.05, N = C:30, R:30, flx-C:29, flx-R:30). (**E**) The percentage of freezing in the fear condition test (2-way ANOVA, stress × durg: p = 0.25; drug: p < 0.001; stress: p < 0.01, N = C:30, R:30, flx-C:29, flx-R:27). (**F**) The number of Brdu^+^ cells (2-way ANOVA, stress × durg: p = 0.62; drug: p < 0.01; stress: p = 0.09, N = C:4, R:4, flx-C:3, flx-R:3). (**G**,**H**) Changes of ATP concentration level in the PFC (**G**) (2-way ANOVA, stress × durg: p = 0.945; drug: p < 0.05; stress: p = 0.436, N = 5 for each group) and hippocampus (**H**) (2-way ANOVA, stress × durg: p < 0.01; drug: p = 0.25; stress: p = 0.73, N = 5 for each group). The asterisks in the figures showed the significant interactions of subgroups detected by post-hoc tests (Tukey Honest Significant Differences): *P < 0.05, **P < 0.01, ***P < 0.001. Data are presented as mean ± SEM.

The 24-hour restraint mice showed depressive-like behaviors and we wondered if these behaviors could be reversed with antidepressant. Fluoxetine (flx) is a classical serotonin selective reuptake inhibitor (SSRI) antidepressant and is widely used for therapy of depression^35^. It is also effective in the treatment of PTSD^36^. We administrated fluoxetine to mice for 5 weeks (Fig. 5A) since 2 days after the restraint and conducted similar behavioral tests mentioned above. Firstly, the sucrose preference of the restraint mice treated with fluoxetine was significantly enhanced (Fig. 5B). Secondly, the latency to first immobility and duration of immobility in the forced swim test were also increased in the restraint mice treated with fluoxetine (Fig. 5C,D). Thirdly, the decreased freezing time in restraint mice was also reversed by fluoxetine treatment (Fig. 5E). In addition, after five weeks fluoxetine treatment, increased generation of new granule neurons were found in DG of the restraint mice (Fig. 5F), similar to findings of previous researches that chronic fluoxetine treatment could increase neurogenesis in adult hippocampus^37^. We further inspected the ATP abundance in the brain, as previous research suggested low ATP abundance in the brain was associated with depressive-like behaviors^38^. Notably, we found the concentration of ATP was decreased in PFC (student t-test, p < 0.05) and hippocampus (student t-test, p < 0.05) in the restraint mice compared to controls and this decrease could be reversed with fluoxetine treatment (Fig. 5G,H). In summary, the depression-like phenotypes induced by 24-hour-restraint could be reversed by chronic fluoxetine treatment.

## Discussion

Recent studies suggested being trapped in an earthquake is one of the strongest risks for depression^7^, PTSD^10^ and ASD^1^. And a number of studies showed the psychiatric symptoms after earthquakes were long lasting, even for years^7^. However, there were few studies targeting on the biological mechanism by using animal model to investigate the impact of stressors related to the earthquake. In our study, we developed 24-hour-restraint mice to simulate the situation of being trapped in an earthquake and found striking depressive-like behaviors and molecular changes. Our study is the first attempt to revel the impact of the 24-hour-restraint condition on mouse model. In our mouse model, we found the impaction of 24-hour-restraint stress on behaviors was long-lasting, and some depressive-like changes (like abnormal cognition function revealed by fear conditioning and the decreased glucose uptake in the brain) are protracted, which was also consistent with previous findings that only a few of acute stress reaction appeared in earthquake victims within 48 hours after the earthquake and the major abnormalities occurred in the following week^39^. Furthermore, several brain regions (STR, CTX, SC and IC) displayed increased glucose uptake in short-term group (48 hours after restraint) and decreased glucose uptake in long-term group (35 days after restraint) by small-animal PET scanning. Considering that there is a delay-action stage of response to stress, the hyperactive glucose metabolism in short-term group might be a temporary acute response to the 24-hour-restraint stress, and the hypoactive glucose metabolism of most brain regions (STR, CTX, SC, IC, HIP, THA, CG and MID) in long-term group presented the long-lasting alterations caused by the 24-hour-restraint stress. Decreasing glucose uptake in cortico-limbic circuitry (as we found in L group experiment) was also found in people with major depressive disorder (MDD)^40^ and PTSD^41^. The results suggested that the dysfunction of cortico-limbic structures might impair the modulation of stress response.

In addition, several differentially expressed genes induced by the 24-hour-restraint were verified by qPCR. The up-regulated genes in long-term PFC and hippocampus, including *Drd2*^42^ and *Adora2a*^43^, had already been recognized and targeted for therapies in schizophrenia, suggesting their specific roles in the pathological process of mental disorders. Besides, genes related to myelination (*Mbp*, *Plp1*, *Ugt8a* and *Ernm*) were found decreased in long-term PFC and long-term hippocampus in our study. Expression level of *Plp1* was decreased in MMD patients^44^ and expression of *Mbp* was reduced in schizophrenia patients^45^. Moreover, a previous microarray study also found genes involved in myelination or structural components of myelin were down-regulated in MDD patients^46^. Although we found several signaling pathways and differentially expressed genes (DEGs) might be involved in the response to the 24-hour-restraint stress, the underlying mechanisms are multifactorial and complex, and the specific roles of these DEGs in the generation of depressive-like behaviors are still unclear and need further exploration. Future studies may focus on these DEGs and related pathways, aiming to reveal the underlying molecular mechanisms.

Previous studies have proved that adult neurogenesis in hippocampus plays an important role in the pathogenesis of mental disorders^47^,^48^ and the regulation of response to stress^23^,^49^. Attenuation of adult-born hippocampal granule neurons could develop depressive actions^50^,^51^. Chronic stress is known to reduce neurogenesis^52^, while the effect of acute stress is somewhat ambiguous. Some studies suggested that acute restraint stress suppressed the generation of new neurons^53^,^54^, while other works acclaimed no changes of proliferation^55^,^56^. An interesting finding was that the cell proliferation was reduced after immobilization for 1 hour and returned to basal level after one day’s recovery compared with controls^54^, suggesting there exists a quick self-balancing capability of adult neurogenesis in response to acute stress. This may be one of the reasons that there is no significant difference about adult neurogenesis between restraint and control mice in short-term group. A recent study found that there was an increased hippocampal cell proliferation in dorsal hippocampus, not ventral hippocampus after 3 hours immobilization^57^, indicating the different roles of ventral and dorsal hippocampus in modulating response to stress. In contrast, another research showed that acute social defeat stress reduced cell survival of adult newborn hippocampal cells without altering the acute proliferation^58^. In our mice model, 24-hour-restraint did not influence the short-term proliferation and survival of adult-born granule cells but inhibited the long-term proliferation and survival of neural cells. These results indicated that the impact of the 24-hour-restraint stress on neurogenesis is time-lapse rather than immediately reacted on the brain. The complex results of acute stress on adult neurogenesis may attribute to the different manipulations to animals, stressors and animal species, and the specific reasons still need further study.

During the past decades, numerous animal models have been established to investigate the neurobiological process of mood disorders, such as depression^59^,^60^ and PTSD^61^, and also to test novel therapeutic drugs. In this paper, we established a new animal model by using 24-hour-restraint condition. The construction procedure of the 24-hour-restraint stress is simple. After 24-hours-restraint, mice were housed in the regular condition, and the mice displayed long-term depressive-like phenotypes 35 days after the restraint. Furthermore, the depressive-like phenotypes of restraint mice are reversed by chronic administrating SSRI drug fluoxetine. Though the modeling process is very much like to build a PTSD model, the restraint mice showed no anxiety-like behaviors (measured by open field test and elevated plus maze), which is a characteristic feature of PTSD^62^. Instead, core depressive-like behaviors (anhedonia and despair) were observed. However, it is also known that comorbid conditions of PTSD and depression are widespread in disaster survivors^63^,^64^, and the depressive-like phenotypes exist both in the PTSD and depression animal models^59^,^62^. Therefore, the 24-hour-restraint mice may be used for further studies on mood disorders.

## Materials and methods

### Animals

Adult male C57/BL6 mice (12 weeks old) were obtained from Model Animal Research Center (MARC) of Nanjing University (Nanjing, China). Mice were housed in groups (N = 5) in standard cages and allowed to acclimate for 7 days before experiments. The mice were kept on a 12h: 12h cycle (lights on at 7:00 a.m.) in a temperature-controlled room maintained at 25 °C with free access to food and water. In this study, different groups of animals were used for the behavioral tests, the PET scan, the post mortem histology and the microarray analysis. All animal experiments were approved by the University Committee on Animal Care and Use of Shanghai Jiao Tong University and conducted in accordance with the approved guidelines.

### Procedure of the 24-hour restraint

The mouse was placed in a ventilated clear plastic tube (3cm in diameter and 10cm in length) and subjected for 24 hours restraint from 10:00 a.m. to 10:00 a.m. of the next day, kept in dark with a background noise by the air-conditioning vents. The holes (0.5 cm in diameter) in the head and along the sidewall of the tune enabled air flowing. Animals could move head and anterior limb, but the body and hindquarters were not able to move or turn around. During the restraint, the animals had no access to food and water. Once the restraint ended, mice were put back to their home cages immediately, with access to food and water freely. Non-restraint mice (control group) remained in the home cages until behavioral experiments started.

### Drug administration

The fluoxetine hydrochloride (J.Huan Chemical Company, Shanghai, China) was dissolved in drinking water with a light-protected bottle. Solutions were prepared freshly everyday with concentration of 0.08 mg/mL, according to the volume of daily water intake of mice to reach approximately 20 mg/kg per day’s dosing. The treatment of fluoxetine was continued for 5 weeks two days after the 24-hour restraint, and throughout all behavioral tests until the mice were sacrificed.

### Sucrose preference test (SPT)

Sucrose preference test was conducted according to a modified protocol described by Snyder, J.S. *et al*.^50^. Briefly, in the adaptation period, mice were individually housed and provided two bottles, containing water and 2% sucrose solution (Vetec, Sigma-Aldrich), respectively. The positions of two bottles were exchanged each 24 hours. After 3 days of habituation, the sucrose preference was first accessed by a pre-SPT to ensure the animals’ preference of sucrose were normal, and mice with preference of sucrose under 65% were considered as anhedonia and would not be used^65^. Water was removed at 4:00 p.m. the day before the sucrose preference test and after 17 hours water deprivation, mice were given access to two bottles containing water and 2% sucrose solution, respectively. The positions of two bottles were exchanged 12 hours later and the consumption of water or sucrose was measured by weighting the bottles at 24h later. After the 24-hour restraint, SPTs of the short and long term groups were also measured as like as the pre-SPT.

### Forced swimming

The forced swimming test was performed in a clear glass cylinder (19.5cm in height, 14.5cm in diameter), filled with water (25 ± 1 °C). The test lasted for 5min in a dim environment. Latency to abandon was scored as the duration from the time when animal was introduced into the water pool, to the first moment of complete immobility (at least 2 seconds long). Parameters of floating behavior were defined as immobility with only occasional slight movements required for keeping the body balanced and the nose above water^66^.

### Contextual fear-conditioning

All animals were allowed to adapt to the experimental room for at least 1 hour before the test. Each test consisted of a training phase and a testing phase. During the training phase, mice were individually placed into the conditioning chamber and allowed to explore for 2 min. The mice were given three shocks (0.75 mA intensity) at 1-min intervals and then returned to their home cages. Training chambers were cleaned with 75% ethanol solution before and after each trial to avoid any olfactive cues. Twenty-four hours after training, mice were placed back into the same conditioning chamber that was used during training for 5 min, and freezing behavior during the 5 min re-exposure to the fear-conditioning chamber was scored.

### Small-animal positron emission tomography (PET) scanning

The small-animal PET scanning was conducted at 2 days and 35 days after restraint. Mice were treated with intraperitoneal injection of 200–300 μCi of 18-fluoro-6-deoxy-glucose (^18^F-FDG) after 48h fasting. After 60min of ^18^F-FDG uptake, mice were anesthetized with isoflurane. Images were obtained after 10-15min scanning by a prototype-dedicated small-animal positron emission tomography (PET; Trans-PET BioCaliburn LH system) scanner developed at the Xie Laboratory, HUST^67^. More details can be found in the supplementary methods.

### Immunohistochemistry

BrdU (Sigma, USA) was intraperitoneally (i.p.) injected with a dose of 100 mg per kg body weight three times during the 24-hour restraint. Animals were deeply anesthetized, then perfused through the heart with 4% paraformaldehyde (PFA) in PBS (pH 7.4). Brains were removed from the skull, postfixed in 4% PFA for 24h at 4 °C, and dehydration in 30% sucrose in PBS solution for 24–48 h until sunk to the bottom. Coronal brain sections were obtained by a cryostat (CM3050 S, Leica) at 40 μm and every six sections were collected in one well. For BrdU staining, the DNA was denatured for 30 min at 37 °C with 2 M HCl, neutralized by 10 min with 0.1 M borate buffer (pH 8.5), then washed with PBS (pH 7.4) three times for 10min. Sections were blocked by 5% goat serum in PBS solution containing 0.3% TritonX-100 (PBST) to prevent non-specific bindings. Then the sections were incubated with the primary antibodies: rat anti-BrdU (1:500, Abcam), rabbit anti-Ki67 (1:50, Millipore) overnight at 4 °C in a humiliated box. After washed with PBS three times of 10min, secondary antibodies, 488 anti-rat (1:500) and 594 anti-rabbit (1:500) (Invitrogen life technologies), were reacted for 2 hours at room temperature and followed with DAPI (Sigma-Aldrich). The numbers of BrdU- and Ki67- positive cells in DG were counted from images obtained under a Leica confocal microscope.

### Microarray

RNA was extracted from prefrontal cortex and hippocampus tissues using the TRIzol^®^ Reagent (Invitrogen Life Technologies) and clean-up by RNasey Mini Kit (Qiagen p/n 74104). RNA was quantified using a NanoDrop ND-1000 and assessed for integrity and gDNA contamination by denaturing agarose gel electrophoresis. The cDNA was generated and labeled by Quick Amp Labeling Kit, One-Color (Agilent p/n 5190-0442) and then hybridization by using Agilent Gene Expression Hybridization Kit (Agilent p/n 5188-5242). Samples were scanning with Agilent Microarray Scanner (Agilent p/n G2565BA). Each mouse was run as an individual to preserve the within-group variability (N = 3 mice per group). Quantile normalization and subsequent data processing were performed using the GeneSpring GX v11.5.1 software package (Agilent Technologies). Probes that passed quality control were filtered for expression before statistical tests (18 948 probes). Pairwise comparisons between conditions were performed with student’s t-test, differentially expressed genes were considered with p < 0.05 and fold change>2 based on the Volcano Plot. Significant differential expressed genes were subjected to pathway analysis and GO analysis based on Genespring to identify their specific biological process or functions.

### Real-Time quantitative PCR (qPCR) verification

Total RNA was purified from the mouse PFC and hippocampus using the PureLink™ RNA Mini Kit (Invitrogen, USA) and reverse transcribed into cDNA using the PrimeScript™ RT reagent Kit (Takara Bio Inc., Japan). The transcripts were amplified using the ABI 7500 Real-Time PCR system (Applied Biosystems) by SYBR™ Fast qPCR Mix (Takara Bio Inc., Japan). Fold changes of expression of genes of interest were normalized to GAPDH endogenous reference gene and then normalized to control samples, and calculated using the ΔΔCt method^68^. The primers used for qPCR were shown as following. Adenosine A_2A_ receptor (*Adora2a*): 5′-CTGCAGAACGTCACCAACTT-3′ (forward) and 5′-CCATTGTACCGGAGTGGAAT-3′ (reverse); Dopamine receptor D2 (*Drd2*): 5′-ACTCAAGGGCAACTGTACCC-3′ (forward) and 5′-TAGACCGTGGTGGGATGGAT-3′ (reverse); Dopamine receptor D1 (*Drd1a*): 5′-CAGATCGGGCATTTGGAGAGA-3′ (forward) and 5′-GGTCCCTAGATTCCCCAAGG-3′ (reverse); 5-hydroxytryptamine (serotonin) receptor 1D (*Htr1d*): 5′-CGTGGAATAGCTGCTGAGTT-3′ (forward) and 5′-TGGAAGCTCTGAGGTGTTTG-3′ (reverse); Neuromedin-B receptor (*Nmbr*): 5′-TGGGTGGTCTCTGTGTTGTT-3′ (forward) and 5′-CCGTGTCTCCATCTGCTTT-3′ (reverse); Myelin basic protein (*Mbp*): 5′-CAGCACCACTCTTGAACACC-3′ (forward) and 5′-GTCCCATTGTTCTGGATCGC-3′ (reverse); Proteolipid protein (myelin) 1 (*Plp1*): 5′-TGTTGTATGGCTCCTGGTGT-3′ (forward) and 5′-ACGCAGCAATAAACAGGTGG-3′ (reverse); UDP galactosyltransferase 8A (*Ugt8a*): 5′-AAGGACGCGCTATGAAGTCT-3′ (forward) and 5′-GCCGATGCTAGTGTCTTGAA-3′ (reverse); Ermin, ERM-like protein (*Ermn*): 5′-TCCGAGAAGGGCATCCGT-3′ (forward) and 5′-AACCCCAGCCATTCGATTTC-3′ (reverse); Glyceraldehyde-3-phosphate dehydrogenase (*Gapdh*): 5′-TGACGTGCCGCCTGGAGAAAC-3′ (forward) and 5′-CCGGCATCGAAGGTGGAAGAG-3′ (reverse).

### ATP level determination

The extraction of ATP from freshly tissues was performed according to a phenol–TE extraction protocol^69^. The ATP levels were determined using a bioluminescent ATP assay kit (FF2000, Promega) following the manufacturer’s instructions.

### Statistical analysis

All results are presented as means ± SEM. For all experiments, student’s t test, two-way or three-way ANOVAs with or without repeated-measure were applied as appropriate. Significant interactions were further detected by unpaired t-test/two-way ANOVA + Holm-Bonferroni correction or Tukey Honest Significant Differences test. All data were analyzed with R.

## Additional Information

**How to cite this article**: Chu, X. *et al*. 24-hour-restraint stress induces long-term depressive-like phenotypes in mice. *Sci. Rep.* **6**, 32935; doi: 10.1038/srep32935 (2016).

## Supplementary Material

Supplementary Information
