# Supplementary material for: 24-hour-restraint stress induces long-term depressive-like phenotypes in mice
Source: Sci Rep. 2016 Sep 9;6:32935. doi: 10.1038/srep32935 (PMC5016966; doi:10.1038/srep32935)
Supplement: Supplementary Information [file srep32935-s1.pdf]

24-hour-restraint stress induces long-term depressive-like phenotypes in mice

Xixia Chu, Ying Zhou, Zhiqiang Hu, Jingyu Lou, Wei Song, Jing Li, Xiao Liang, Chen Chen, Shuai Wang, Beimeng Yang, Lei Chen, Xu Zhang, Jinjing Song, Yujie Dong, Shiqing Chen, Lin He, Guoqing Xie, Xiaoping Chen, Weidong Li

Supplementary information

Supplementary Figures

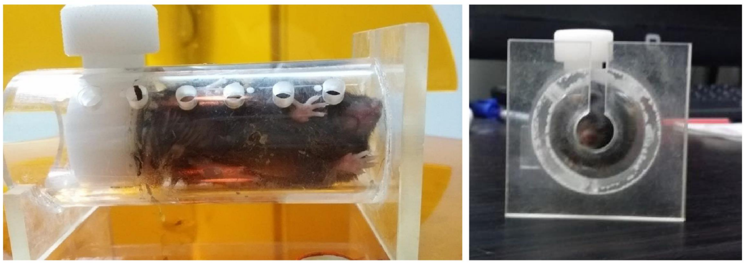

Figure S1. The apparatus of the 24-hour-restraint. The restraint apparatus is a ventilated clear plastic tube (3cm in diameter×10cm). The holes (0.5 cm in diameter) in the head and along the sidewall of the tune enabled air flowing and keep warm as well. Animals could move head and anterior limb, but the body and hindquarters were not able to move or turn around.

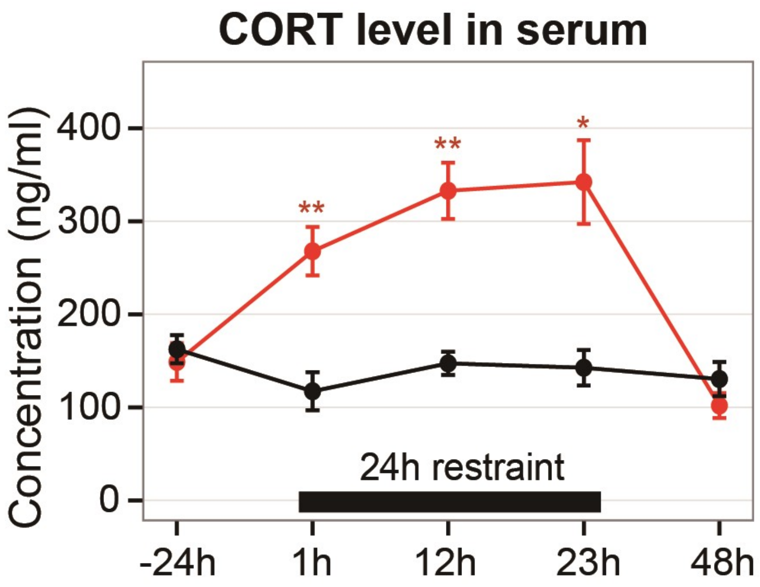

Figure S2. The changes of serum corticosterone (CORT) level. The serum CORT levels were tested 24 hours before the restraint (-24h), within the restraint (1h, 12h and 23h) and after the restraint (48h). The restraint and control groups were displayed in red and black respectively. Two-way ANOVA with repeated-measure (N=7 for each group) suggested CORT levels between two groups were different ($p<0.001$). Significant interactions were detected by additional unpaired t-tests followed by correction with Holm-Bonferroni method. Data are presented as mean ±SEM, *P< 0.05, ** P< 0.01.

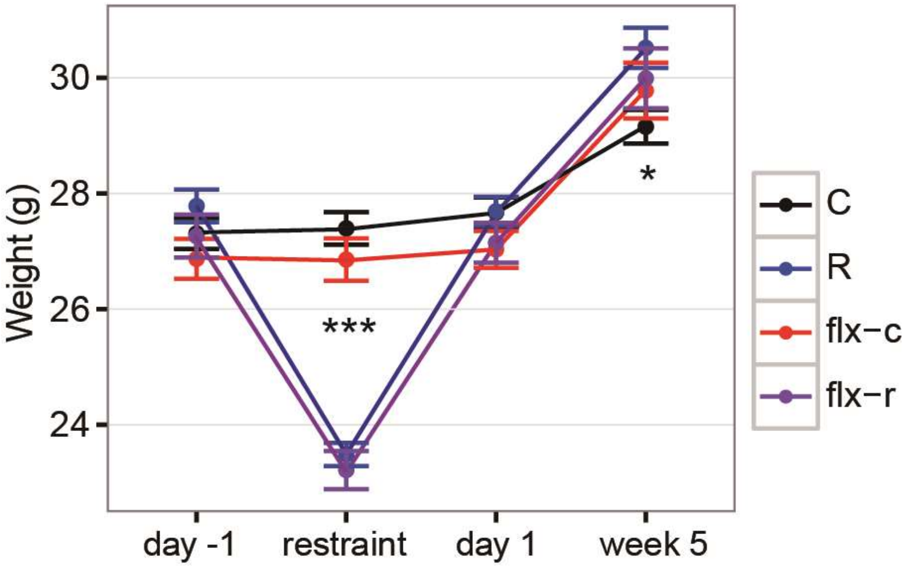

Figure S3. Changes of body weight. The weight of mice was measured 24 hours before restraint (Day-1), immediately after restraint was ended (restraint), 1 day after restraint (Day 1), and 5 weeks after the restraint (Week 5). Three-way ANOVA with repeated-measure suggested body weights of mice with or without restraint were different ($p<0.05$). Significant interactions of time points and stress/restraint were detected by two-way ANOVAs followed by correction with Holm-Bonferroni method. Data are presented as mean ±SEM, *$P< 0.05$, *** $P< 0.001$.

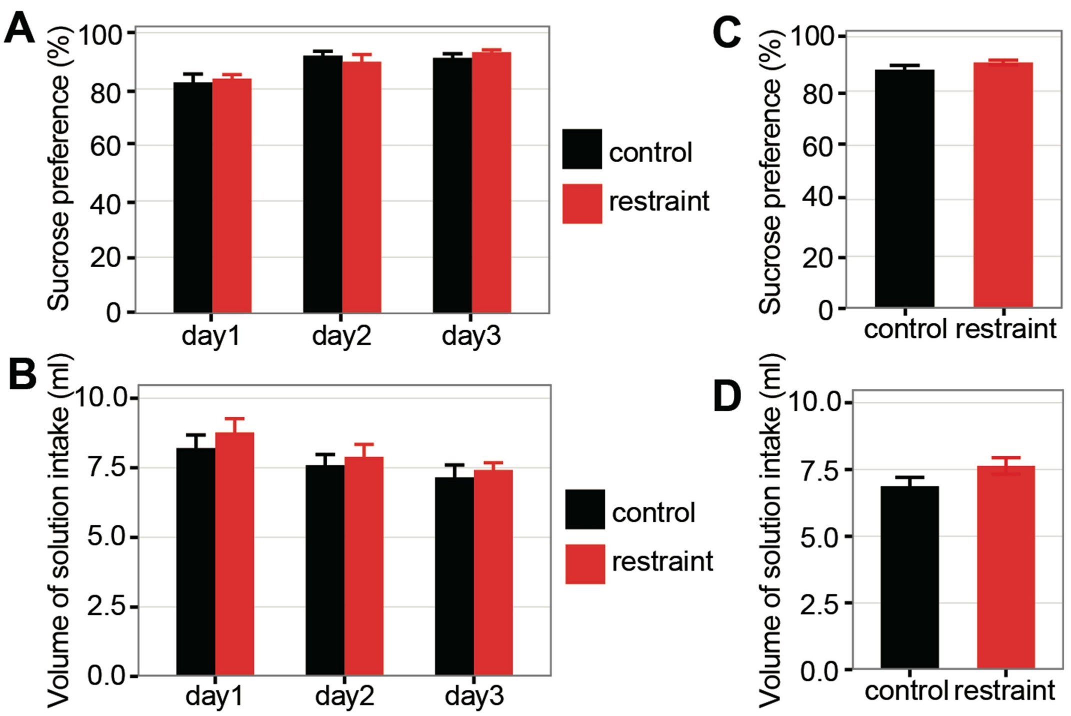

Figure S4. Sucrose preference adaptation and pre-SPT before the 24-hour-restraint of S group showed no difference between control and restraint mice. A. Sucrose preference (%) in the 3-day adaption (two-way ANOVA, p=0.788). **B.** Total consumption (water + sucrose) in 3-day adaptation (two-way ANOVA, p=0.278). **C.** Sucrose consumption percentage in the 24-hour sucrose preference test before restraint (t-test, p=0.103). **D.** Total consumption in the 24-hour sucrose preference test before restraint (t-test, p=0.163). N=24 vs. 25, respectively. Data are presented as mean ±SEM.

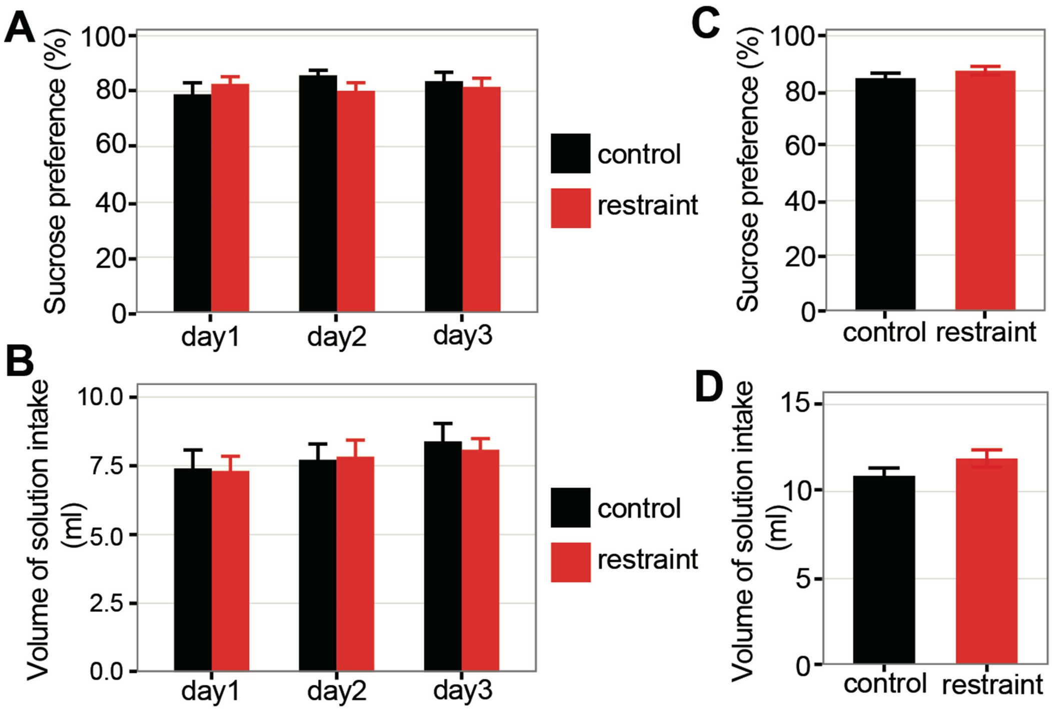

Figure S5. Sucrose preference adaptation and pre-SPT before 24-hour-restraint of L group showed no difference between control and restraint mice. A. Sucrose consumption in the 3-day adaption (two-way ANOVA, p=0.61). **B.** Total consumption (water + sucrose) in the 3-day adaptation (two-way ANOVA, p=0.84). **C.** Sucrose consumption percentage in the 24-hour sucrose preference test before restraint (t-test, p=0.24). **D.** Total consumption in the 24-hour sucrose preference test before restraint (t-test, p=0.14). N=25 vs. 27, respectively. Data are presented as mean ±SEM.

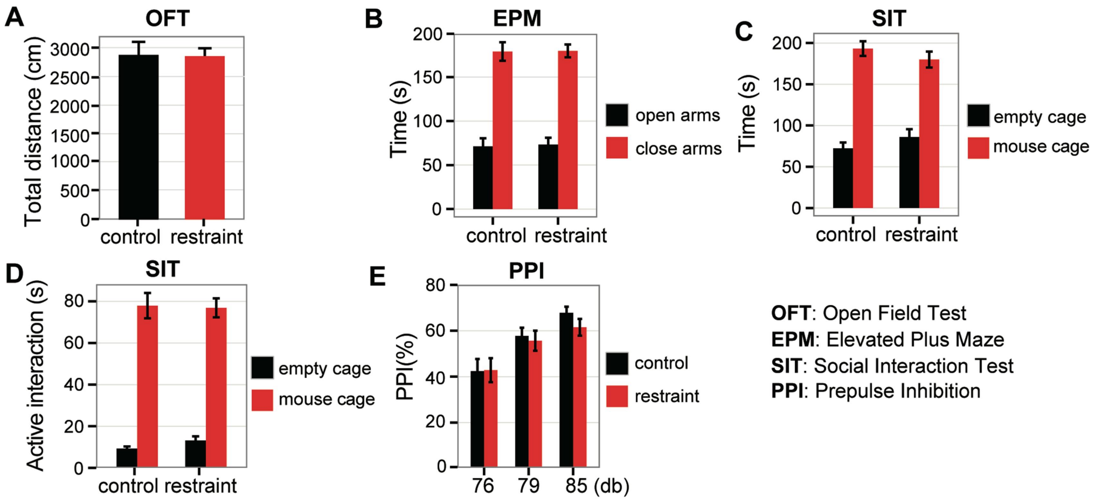

Figure S6. Behavioral experiments without significant differences of the short-term group. A. The total distances in the open field experiment (p=0.937, N=15 vs. 15). **B.** The dwell time between in the open arms and in the close arms in the elevated plus maze experiment (p=0.860 for open arm comparison, p=0.957 for close arm comparison, N=15 vs. 15). **C**. Time in mouse cage and empty cage in the sociability test (p=0.272 and 0.325, N=15 vs. 15). **D.** The sniffing time between in mouse cage cell and empty cage cell in the sociability test (p=0.101 and 0.890, N=15 vs. 15). **E.** The %PPI in the prepulse inhibition test (p=0.952, 0.707 and 0.175 for 76db, 79db and 85db respectively, N=15 vs. 15). Data are presented as mean ±SEM.

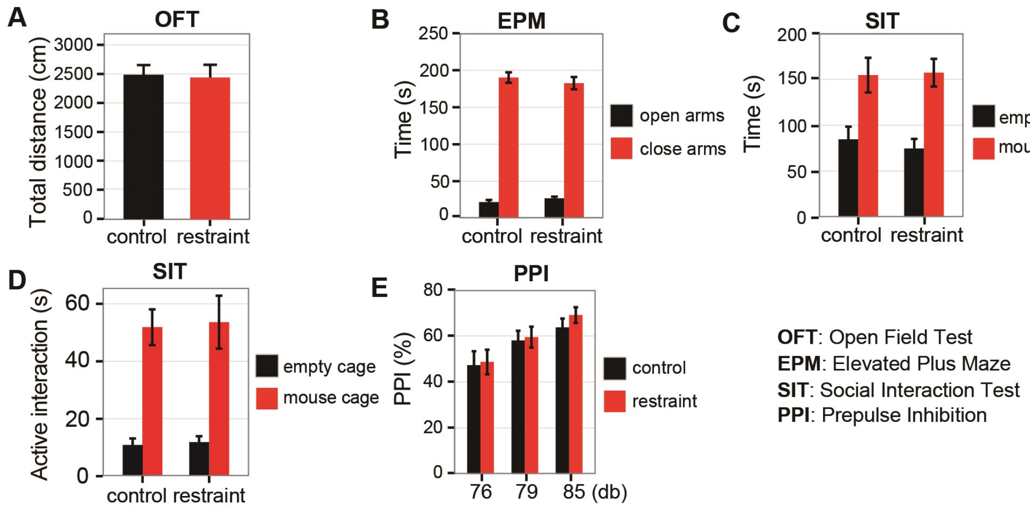

Figure S7. Behavioral experiments without significant differences of the long-term group. A. The total distances in the open field experiment (p=0.866, N=15 vs. 15). **B.** The dwell time between in the open arms and in the close arms in the elevated plus maze experiment (p=0.112 for open arm comparison, p=0. 170 for close arm comparison, N=15 vs. 15) **C.** The dwell time between in mouse cage cell and empty cage cell in the sociability test (p=0.913 and 0.563, N=13 vs. 15). **D.** The sniffing time between in mouse cage cell and empty cage cell in the sociability test (p=0.895 and 0.769, N=13 vs. 15). **E.** The %PPI in the prepulse inhibition test (p=0.865, 0.815 and 0.302 for 76db, 79db and 85db respectively, N=15 vs. 15). Data are presented as mean ±SEM.

Supplementary methods

Corticosterone assay

To measure the level of CORT, 100μl blood samples were collected from tail vein at 5 time points (24 hours before restraint, 1, 12, 23 and 48 hours after the onset of restraint). Blood samples were centrifuged at 1,000g for 10minutes, and serum was collected. The level of CORT was measured by radioimmunoassay using the corticosterone kit (MP Biomedicals, USA).

Open Field test (OFT)

All animals were allowed to adapt to the experimental room for at least 1 hour before the test. Open field test (OFT) is commonly used for measuring the exploratory behavior and general activity of animals. Mice were allowed to freely explore the chamber for 20 min in a square plexiglass box (27.5×27.5×18 cm). The total distance of movement was recorded by software of Med Associates inc.

Elevated Plus-maze test (EMP)

The apparatus consisted of consisted of two open arms (30×8 cm) and two closed arms (30×8 cm, surrounded by 15 cm-high walls), positioned opposite each other. Mice were placed in the center with its nose directed toward the closed arm. Each session lasted 5 min and maze was cleaned with alcohol after each test. Behavior of each mouse was continuously videotaped by a video camera placed above the

apparatus.

Social Interaction test (SIT)

The apparatus is a plexiglass box (60×40×50 cm) consisted of three chambers divided by plexiglass walls with holes in the middle to allow animals to move between chambers. Inverted steel cages (8 in diameter × 10 cm) were placed in each side of the end chambers. The test consists of two sessions. In the first session, mouse was placed in the central chamber and allowed to habituate to the apparatus for a 10 min. In the second session, a stimulus juvenile male mouse (4-5 weeks) was placed in the inverted cage used for the social compartment and the test mouse was palced in the middle chanmer to explore for 5 min. Behavioral tests were recorded by a video camera, and the EthoVision XT video tracking system (Noldus).

Prepulse Inhibition test (PPI)

The test had a total of 90 trials by The SR-LAB™ Startle Response System. To evaluate the startle response, each of the first 10 trials is consisted of a 40 ms 120 dB "pulse alone" startle stimuli. The rest of the 80 trials are consisted of random delivery of: 20 "pulse alone" startle stimuli, 30 "pre" stimuli (at 76, 79 and 85dB), and 30 "pre-pulse" trials that consisted of a single 120 dB pulse preceded by a 20 ms pre pulse. PPI was defined as the percent reduction in startle magnitude in each prepulse condition compared to the magnitude of response in the pulse-alone condition.

PET scanning

After a PET scan with the trans-PET® BioCaliburn® LH system, PET images were reconstructed by use of 3D OSEM method, with the voxel size of $0.5 \times 0.5 \times 0.5$ mm^3. This trans-PET® system has the radial, tangential and axial resolutions of 0.95 mm FWHM, 1.05 mm FWHM and 1.01 mm FHWM, respectively, at the center of the AFOV. The absolute sensitivity measured at the center of the scanner is 2.04% with an energy window of 350-650 keV and coincidence timing window of 5 ns. The noise equivalent count-rate reaches a peak value of 62 kcps at 28 MBq for the mouse-sized phantom. The scatter fraction is 8.4% for the mouse-sized phantom. For the Derenzo phantom study, the 1.0 mm diameter rods can be clearly resolved [1].

In this work, the volume of the VOIs ranges from 5 mm^3 to 150 mm^3. Among the brain areas, the inferior colliculus has the smallest VOI of 5.1 mm^3. Hence, it is possible to differentiate between different brain areas according to the Derenzo phantom study with this PET system. In addition, to obtain the quantitative data of each VOI, an imaging processing method is employed. An ^{18}FDG uptake was calculated in each VOI of each subject by use of an individual normalized parametric images and an in-house generated VOI-template[2]. The VOI template for whole-brain quantification of the ^{18}FDG-PET signal was created by use of the brain atlas generated from the MRI or immunohistochemical images[3]. The PET scan for each subject in the study was aligned to the ^{18}FDG-mouse-template with the VOI template. The percent injected dose per cubic centimeter of tissue (%ID/cc tissue) was obtained

for each VOI by dividing the mean ^{18}FDG activities of the injected dose.

As the brain areas are small, the quantitative imaging should be improved by developing image analysis methods based on understanding the physical factors affecting the precision, such as spatial resolution, noise, and some unclear ones. The partial volume effects may result that the small objects appear to be smeared out and have lower activity concentration in the reconstructed PET image, while spill-over effects lead to higher activity concentration. How these factors affecting the PET imaging analysis and the smallest areas that can be quantitated by a PET scanner need to be further studied.

References:

1. Wang, L. et al. Performance evaluation of the Trans-PET® BioCaliburn® LH system: a large FOV small-animal PET system. Phys Med Biol 60, 137–50 (2015).
2. Mirrione, M. M. et al. Increased metabolic activity in the septum and habenula during stress is linked to subsequent expression of learned helplessness behavior. Front Hum Neurosci 8, 29 (2014).
3. Ma, Y. et al. In Vivo 3D Digital Atlas Database of the Adult C57BL/6J Mouse Brain by Magnetic Resonance Microscopy. Front Neuroanat 2, 1 (2008).

Table S1 Enriched KEGG pathways.

Tissue	Time	Enriched pathways	p-value	Involved genes
prefrontal Cortex	35d	Antigen processing and presentation	8.37E-05	CD8B1//H2-Q10//HSPA1A//HSPA1B
		Protein processing in endoplasmic reticulum	0.014	HSPA1A//HSPA1B//PDIA4
		Adipocytokine signaling pathway	0.018	IRS2//PRKAG2
		Endocytosis	0.028	H2-Q10//HSPA1A//HSPA1B
		MAPK signaling pathway	0.041	CACNA2D1//HSPA1A//HSPA1B
		Neuroactive ligand-receptor interaction	2.86E-06	ADORA2A//DRD1A//DRD2//HTR1D//NMBR//NTS
		Dopaminergic synapse	0.001	DRD1A//DRD2//KCNJ5
		Gap junction	0.007	DRD1A//DRD2
		Serotonergic synapse	0.016	HTR1D//KCNJ5
		Calcium signaling pathway	0.03	ADORA2A//DRD1A
	2d	T cell receptor signaling pathway	0.009	CD8B1//IL5//MAPK12//NFATC1//PTPRC
		Notch signaling pathway	0.02	DLL1//DLL4//KAT2A
		Inositol phosphate metabolism	0.035	MIOX//PLCZ1//TPI1
		Pancreatic secretion	0.035	ATP2B4//CELA2A//PLA2G12A//SCT
		Neuroactive ligand-receptor interaction	0.037	ADRA2B//ADRB3//CHRNA2//GABRB3//MC3R//NMUR2//NTS//SCT
		Cardiac muscle contraction	<0.001	ACTC1//COX8B//MYL3//TNNC1//TNNI3//TNNT2//TPM1
		Calcium signaling pathway	0.047	ADCY3//MYLK3//PLN//TNNC1//TRHR
Hippocampus	35d	MAPK signaling pathway	0.001	DUSP5//FGFR2//HSPA1A//HSPA1B//IKBKG//MYC//TRAF6
		Endocytosis	0.008	FGFR2//H2-M2//HSPA1A//HSPA1B//TRAF6
		B cell receptor signaling pathway	0.01	IKBKG//INPP5D//PTPN6
		Antigen processing and presentation	0.011	H2-M2//HSPA1A//HSPA1B
		Hedgehog signaling pathway	0.032	WNT3//WNT7B
		Gap junction	0.001	DRD1A//GUCY1B2//PRKG2//TUBA1C//TUBA3A
		Neuroactive ligand-receptor interaction	0.001	ADORA2A//DRD1A//F2RL1//GABRR1//HTR1D//LEPR//SCT//TACR1//TRH
		Antigen processing and presentation	0.028	H2-Q1//HSP90AB1//KLRC2
		Hippo signaling pathway	0.039	BMP6//CRB2//CTGF//WNT6
	2d	Fatty acid metabolism	0.027	ACSL3//ALDH7A1
		Hedgehog signaling pathway	0.028	BMP4//ZIC2
		Arginine and proline metabolism	0.037	ALDH7A1//P4HA1

Enrichments from up-regulated DEGs and down-regulated DEGs are shown in “red” and “green” respectively.

Table S2 Enriched GO terms of DEGs.

A. Enriched GO terms of up-regulated DEGs in long-term PFC.

Term	Count	PValue	Genes	Fold Enrichment	Bonferroni	Benjamini	FDR
GO:0007631~feeding behavior	4	2.70E-05	CALCA, ADORA2A, DRD2, DRD1A	62.68973472	0.014581416	0.014581416	0.039274176
GO:0007188~G-protein signaling, coupled to cAMP nucleotide second messenger	4	3.39E-05	CALCA, ADORA2A, DRD2, DRD1A	58.13048128	0.018282456	0.009183395	0.049332702
GO:0007187~G-protein signaling, coupled to cyclic nucleotide second messenger	4	4.40E-05	CALCA, ADORA2A, DRD2, DRD1A	53.28627451	0.023696227	0.007961967	0.064112691
GO:0045761~regulation of adenylate cyclase activity	4	4.40E-05	CALCA, ADORA2A, DRD2, DRD1A	53.28627451	0.023696227	0.007961967	0.064112691
GO:0019933~cAMP-mediated signaling	4	4.62E-05	CALCA, ADORA2A, DRD2, DRD1A	52.41272903	0.024888871	0.006281149	0.067379416
GO:0031279~regulation of cyclase activity	4	4.86E-05	CALCA, ADORA2A, DRD2, DRD1A	51.56736243	0.026119231	0.005279295	0.070753527
GO:0051339~regulation of lyase activity	4	4.86E-05	CALCA, ADORA2A, DRD2, DRD1A	51.56736243	0.026119231	0.005279295	0.070753527
GO:0030817~regulation of cAMP biosynthetic process	4	5.60E-05	CALCA, ADORA2A, DRD2, DRD1A	49.18733032	0.030041045	0.005070688	0.081536369
GO:0019935~cyclic-nucleotide-mediated signaling	4	5.86E-05	CALCA, ADORA2A, DRD2, DRD1A	48.44206774	0.031426656	0.004551193	0.085356188

GO:0030814~regulation of cAMP metabolic process	4	6.13E-05	CALCA, ADORA2A, DRD2, DRD1A	47.7190518	0.032852127	0.004166779	0.089291447
GO:0030802~regulation of cyclic nucleotide biosynthetic process	4	6.99E-05	CALCA, ADORA2A, DRD2, DRD1A	45.67394958	0.037371644	0.004223043	0.101805776
GO:0030808~regulation of nucleotide biosynthetic process	4	6.99E-05	CALCA, ADORA2A, DRD2, DRD1A	45.67394958	0.037371644	0.004223043	0.101805776
GO:0044057~regulation of system process	5	7.36E-05	CALCA, GSX2, ADORA2A, DRD2, DRD1A	19.88293825	0.039305027	0.004001804	0.107176696
GO:0030799~regulation of cyclic nucleotide metabolic process	4	7.92E-05	CALCA, ADORA2A, DRD2, DRD1A	43.79693795	0.042263646	0.003918008	0.115416112
GO:0051966~regulation of synaptic transmission, glutamatergic	3	8.52E-05	ADORA2A, DRD2, DRD1A	199.8235294	0.045379667	0.003862652	0.124120704
GO:0006140~regulation of nucleotide metabolic process	4	8.59E-05	CALCA, ADORA2A, DRD2, DRD1A	42.62901961	0.045736564	0.003594716	0.12511946
GO:0001975~response to amphetamine	3	1.35E-04	ADORA2A, DRD2, DRD1A	159.8588235	0.071081297	0.005252873	0.19699126
GO:0031645~negative regulation of neurological system process	3	1.97E-04	CALCA, DRD2, DRD1A	133.2156863	0.101674821	0.007122723	0.286334092
GO:0014075~response to amine stimulus	3	2.44E-04	ADORA2A, DRD2, DRD1A	119.8941176	0.124511529	0.008276392	0.354976359
GO:0010243~response to organic nitrogen	3	2.44E-04	ADORA2A, DRD2, DRD1A	119.8941176	0.124511529	0.008276392	0.354976359

GO:0003013~circulatory system process	4	2.75E-04	CALCA, NTS, ADORA2A, DRD2	28.80339163	0.139182199	0.008777277	0.399998732
GO:0008015~blood circulation	4	2.75E-04	CALCA, NTS, ADORA2A, DRD2	28.80339163	0.139182199	0.008777277	0.399998732
GO:0031644~regulation of neurological system process	4	2.90E-04	CALCA, ADORA2A, DRD2, DRD1A	28.29359708	0.146125621	0.008737779	0.42156818
GO:0019932~second-messenger-mediated signaling	4	3.29E-04	CALCA, ADORA2A, DRD2, DRD1A	27.09471585	0.164266325	0.00940003	0.478737417
GO:0007270~nerve-nerve synaptic transmission	3	4.83E-04	ADORA2A, DRD2, DRD1A	85.63865546	0.231357137	0.013070274	0.701210284
GO:0043279~response to alkaloid	3	5.55E-04	ADORA2A, DRD2, DRD1A	79.92941176	0.260959331	0.014296925	0.8054474
GO:0010578~regulation of adenylate cyclase activity involved in G-protein signaling	3	6.32E-04	CALCA, ADORA2A, DRD1A	74.93382353	0.291317913	0.015530334	0.916656665
GO:0007189~activation of adenylate cyclase activity by G-protein signaling pathway	3	6.32E-04	CALCA, ADORA2A, DRD1A	74.93382353	0.291317913	0.015530334	0.916656665
GO:0010579~positive regulation of adenylate cyclase activity by G-protein signaling pathway	3	6.32E-04	CALCA, ADORA2A, DRD1A	74.93382353	0.291317913	0.015530334	0.916656665
GO:0030182~neuron differentiation	5	0.001006849	GSX2, ADORA2A, DRD2, EN2, DRD1A	10.01621701	0.422478078	0.023587317	1.457478185
GO:0007190~activation of adenylate cyclase activity	3	0.001037799	CALCA, ADORA2A, DRD1A	58.48493544	0.432147728	0.023303105	1.501965506
GO:0031281~positive regulation of cyclase	3	0.00108894	CALCA, ADORA2A, DRD1A	57.09243697	0.44777305	0.023471992	1.575435653

activity		1					
GO:0045762~positive regulation of adenylate cyclase activity	3	0.00108894<br>1	CALCA, ADORA2A, DRD1A	57.09243697	0.44777305	0.023471992	1.575435653
GO:0051349~positive regulation of lyase activity	3	0.00108894<br>1	CALCA, ADORA2A, DRD1A	57.09243697	0.44777305	0.023471992	1.575435653
GO:0014070~response to organic cyclic substance	3	0.00108894<br>1	ADORA2A, DRD2, DRD1A	57.09243697	0.44777305	0.023471992	1.575435653
GO:0048167~regulation of synaptic plasticity	3	0.00142076<br>4	ADORA2A, DRD2, DRD1A	49.95588235	0.539234912	0.029362857	2.050888783
GO:0050880~regulation of blood vessel size	3	0.00142076<br>4	CALCA, NTS, ADORA2A	49.95588235	0.539234912	0.029362857	2.050888783
GO:0035150~regulation of tube size	3	0.00142076<br>4	CALCA, NTS, ADORA2A	49.95588235	0.539234912	0.029362857	2.050888783
GO:0003018~vascular process in circulatory system	3	0.00154082<br>3	CALCA, NTS, ADORA2A	47.95764706	0.568460573	0.030646372	2.222388572
GO:0008227~amine receptor activity	3	0.00158539<br>8	DRD2, HTR1D, DRD1A	47.00943396	0.1164074	0.1164074	1.664328376
GO:0043176~amine binding	3	0.00209448	DRD2, HTR1D, DRD1A	40.8442623	0.15086814	0.07851649	2.193410917
GO:0048871~multicellular organismal	3	0.00308096	TEX15, DRD2, DRD1A	33.77299089	0.813946171	0.05829329	4.397755673

homeostasis		7					
GO:0021537~telencephalon development	3	0.003166823	GSX2, DRD2, DRD1A	33.30392157	0.822477345	0.057867036	4.517684957
GO:0043085~positive regulation of catalytic activity	4	0.00325975	CALCA, ADORA2A, DRD2, DRD1A	12.24971828	0.831271726	0.05759063	4.647334979
GO:0051968~positive regulation of synaptic transmission, glutamatergic	2	0.00352863	ADORA2A, DRD1A	532.8627451	0.854343532	0.060253671	5.021547078
GO:0042592~homeostatic process	5	0.004065992	CALCA, TEX15, ADORA2A, DRD2, DRD1A	6.843271555	0.891443327	0.067037331	5.765322123
GO:0048666~neuron development	4	0.004469459	ADORA2A, DRD2, EN2, DRD1A	10.94923449	0.91295367	0.071309023	6.320200347
GO:0050801~ion homeostasis	4	0.004512445	CALCA, ADORA2A, DRD2, DRD1A	10.91186509	0.914978249	0.0699301	6.379139055
GO:0021853~cerebral cortex GABAergic interneuron migration	2	0.004702244	DRD2, DRD1A	399.6470588	0.923370037	0.070764753	6.638958126
GO:0021894~cerebral cortex GABAergic interneuron development	2	0.004702244	DRD2, DRD1A	399.6470588	0.923370037	0.070764753	6.638958126
GO:0021830~interneuron migration from the subpallium to the cortex	2	0.004702244	DRD2, DRD1A	399.6470588	0.923370037	0.070764753	6.638958126

GO:0060158~activation of phospholipase C activity by dopamine receptor signaling pathway	2	0.004702244	DRD2, DRD1A	399.6470588	0.923370037	0.070764753	6.638958126
GO:0044093~positive regulation of molecular function	4	0.005094474	CALCA, ADORA2A, DRD2, DRD1A	10.44828912	0.938183491	0.07440805	7.173763574
GO:0004952~dopamine receptor activity	2	0.005632308	DRD2, DRD1A	332.2	0.356325337	0.136579933	5.799609945
GO:0021826~substrate-independent telencephalic tangential migration	2	0.005874562	DRD2, DRD1A	319.7176471	0.959686833	0.083126594	8.228941996
GO:0003091~renal water homeostasis	2	0.005874562	ADORA2A, DRD2	319.7176471	0.959686833	0.083126594	8.228941996
GO:0021843~substrate-independent telencephalic tangential interneuron migration	2	0.005874562	DRD2, DRD1A	319.7176471	0.959686833	0.083126594	8.228941996
GO:0030146~diuresis	2	0.005874562	ADORA2A, DRD2	319.7176471	0.959686833	0.083126594	8.228941996
GO:0030147~natriuresis	2	0.005874562	ADORA2A, DRD2	319.7176471	0.959686833	0.083126594	8.228941996
GO:0051590~positive regulation of neurotransmitter transport	2	0.005874562	ADORA2A, DRD2	319.7176471	0.959686833	0.083126594	8.228941996
GO:0050804~regulation of synaptic transmission	3	0.00601635	ADORA2A, DRD2, DRD1A	23.97882353	0.962702102	0.082908335	8.419537336

		8					
GO:0051241~negative regulation of multicellular organismal process	3	0.006016358	CALCA, DRD2, DRD1A	23.97882353	0.962702102	0.082908335	8.419537336
GO:0030424~axon	3	0.006173201	CALCA, ADORA2A, DRD2	23.37196262	0.275302783	0.275302783	5.838852183
GO:0032101~regulation of response to external stimulus	3	0.006371524	CALCA, ADORA2A, DRD2	23.28041119	0.969303003	0.08544972	8.895317197
GO:0051969~regulation of transmission of nerve impulse	3	0.006859709	ADORA2A, DRD2, DRD1A	22.41011545	0.976515699	0.089522032	9.545531972
GO:0014002~astrocyte development	2	0.007045585	ADORA2A, DRD1A	266.4313725	0.978793223	0.089704682	9.791961814
GO:0050891~multicellular organismal water homeostasis	2	0.007045585	ADORA2A, DRD2	266.4313725	0.978793223	0.089704682	9.791961814
GO:0040012~regulation of locomotion	3	0.00723674	ADORA2A, DRD2, DRD1A	21.79893048	0.980905658	0.089941816	10.0447387
GO:0035240~dopamine binding	2	0.007876929	DRD2, DRD1A	237.2857143	0.460349228	0.142906502	8.024735557
GO:0021892~cerebral cortex GABAergic interneuron differentiation	2	0.008215315	DRD2, DRD1A	228.3697479	0.988844685	0.099274203	11.32847292
GO:0014821~phasic smooth muscle contraction	2	0.00821531	DRD2, DRD1A	228.3697479	0.988844685	0.099274203	11.32847292

		5					
GO:0030432~peristalsis	2	0.008215315	DRD2, DRD1A	228.3697479	0.988844685	0.099274203	11.32847292
GO:0001963~synaptic transmission, dopaminergic	2	0.008215315	ADORA2A, DRD1A	228.3697479	0.988844685	0.099274203	11.32847292
GO:0048878~chemical homeostasis	4	0.008299598	CALCA, ADORA2A, DRD2, DRD1A	8.759387591	0.989349584	0.098081236	11.43823797
GO:0048708~astrocyte differentiation	2	0.009383752	ADORA2A, DRD1A	199.8235294	0.994132292	0.107906426	12.83892299
GO:0051954~positive regulation of amine transport	2	0.009383752	ADORA2A, DRD2	199.8235294	0.994132292	0.107906426	12.83892299
GO:0048148~behavioral response to cocaine	2	0.009383752	DRD2, DRD1A	199.8235294	0.994132292	0.107906426	12.83892299
GO:0060134~prepulse inhibition	2	0.009383752	ADORA2A, DRD2	199.8235294	0.994132292	0.107906426	12.83892299
GO:0007625~grooming behavior	2	0.0105509	DRD2, DRD1A	177.620915	0.996913725	0.118093607	14.32375219
GO:0007610~behavior	4	0.011025213	CALCA, ADORA2A, DRD2, DRD1A	7.89426289	0.997623461	0.120635275	14.92040658
GO:0030104~water homeostasis	2	0.01171675	ADORA2A, DRD2	159.8588235	0.99837677	0.125252079	15.78339327

		8					
GO:0043178~alcohol binding	2	0.012351993	DRD2, DRD1A	151	0.62071037	0.17625234	12.31949552
GO:0032228~regulation of synaptic transmission, GABAergic	2	0.012881328	ADORA2A, DRD2	145.3262032	0.9991463	0.134287731	17.21827169
GO:0021895~cerebral cortex neuron differentiation	2	0.014044611	DRD2, DRD1A	133.2156863	0.999551038	0.142874948	18.62880578
GO:0050433~regulation of catecholamine secretion	2	0.014044611	ADORA2A, DRD2	133.2156863	0.999551038	0.142874948	18.62880578
GO:0042220~response to cocaine	2	0.015206609	DRD2, DRD1A	122.9683258	0.999763901	0.151045955	20.01540681
GO:0050805~negative regulation of synaptic transmission	2	0.015206609	DRD2, DRD1A	122.9683258	0.999763901	0.151045955	20.01540681
GO:0021544~subpallium development	2	0.015206609	GSX2, DRD1A	122.9683258	0.999763901	0.151045955	20.01540681
GO:0007589~body fluid secretion	2	0.015206609	ADORA2A, DRD2	122.9683258	0.999763901	0.151045955	20.01540681
GO:0014073~response to tropane	2	0.015206609	DRD2, DRD1A	122.9683258	0.999763901	0.151045955	20.01540681

GO:0051240~positive regulation of multicellular organismal process	3	0.015370129	ADORA2A, DRD2, DRD1A	14.71093468	0.99978433	0.149849256	20.2087572
GO:0007186~G-protein coupled receptor protein signaling pathway	7	0.016006943	CALCA, ADORA2A, DRD2, RGS9, HTR1D, NMBR, DRD1A	2.980851797	0.999848414	0.152895564	20.95760358
GO:0030900~forebrain development	3	0.016092075	GSX2, DRD2, DRD1A	14.35857696	0.999855396	0.151030236	21.05721501
GO:0051970~negative regulation of transmission of nerve impulse	2	0.016367323	DRD2, DRD1A	114.1848739	0.999875847	0.150856621	21.37847916
GO:0001976~neurological system process involved in regulation of systemic arterial blood pressure	2	0.016367323	CALCA, DRD2	114.1848739	0.999875847	0.150856621	21.37847916
GO:0007268~synaptic transmission	3	0.018151656	ADORA2A, DRD2, DRD1A	13.47124917	0.999953847	0.163289765	23.43175947
GO:0002027~regulation of heart rate	2	0.018684905	CALCA, DRD2	99.91176471	0.999965674	0.165017129	24.03562132
GO:0045776~negative regulation of blood pressure	2	0.018684905	CALCA, DRD2	99.91176471	0.999965674	0.165017129	24.03562132
GO:0009719~response to endogenous stimulus	3	0.019320208	ADORA2A, DRD2, DRD1A	13.03196931	0.999975882	0.167498274	24.74926043

GO:0007242~intracellular signaling cascade	5	0.019363534	CALCA, ADORA2A, DRD2, RGS9, DRD1A	4.367727419	0.999976456	0.165248303	24.79769984
GO:0010648~negative regulation of cell communication	3	0.019716702	DRD2, RGS9, DRD1A	12.89184061	0.999980653	0.165466591	25.19147451
GO:0001964~startle response	2	0.019841775	ADORA2A, DRD2	94.03460208	0.999981952	0.163941938	25.3304663
GO:0001659~temperature homeostasis	2	0.020997367	DRD2, DRD1A	88.81045752	0.999990511	0.170173773	26.60333313
GO:0022029~telencephalon cell migration	2	0.020997367	DRD2, DRD1A	88.81045752	0.999990511	0.170173773	26.60333313
GO:0006939~smooth muscle contraction	2	0.020997367	DRD2, DRD1A	88.81045752	0.999990511	0.170173773	26.60333313
GO:0008306~associative learning	2	0.020997367	DRD2, DRD1A	88.81045752	0.999990511	0.170173773	26.60333313
GO:0051336~regulation of hydrolase activity	3	0.021750766	ADORA2A, DRD2, DRD1A	12.23409364	0.999993763	0.173237557	27.42227662
GO:0021885~forebrain cell migration	2	0.022151681	DRD2, DRD1A	84.13622291	0.999995012	0.173665875	27.85459329
GO:0007212~dopamine receptor signaling	2	0.02330471	DRD2, DRD1A	79.92941176	0.999997378	0.17939548	29.08461199

pathway		9					
GO:0007588~excretion	2	0.024456482	ADORA2A, DRD2	76.1232493	0.999998621	0.184914105	30.2937483
GO:0050806~positive regulation of synaptic transmission	2	0.024456482	ADORA2A, DRD1A	76.1232493	0.999998621	0.184914105	30.2937483
GO:0051971~positive regulation of transmission of nerve impulse	2	0.025606972	ADORA2A, DRD1A	72.6631016	0.999999275	0.190233097	31.48235525
GO:0051588~regulation of neurotransmitter transport	2	0.025606972	ADORA2A, DRD2	72.6631016	0.999999275	0.190233097	31.48235525
GO:0021782~glial cell development	2	0.02675619	ADORA2A, DRD1A	69.50383632	0.999999619	0.195363013	32.65077992
GO:0033267~axon part	2	0.027644356	CALCA, ADORA2A	66.688	0.767240591	0.517548542	23.84238433
GO:0031646~positive regulation of neurological system process	2	0.027904137	ADORA2A, DRD1A	66.60784314	0.9999998	0.200313685	33.79936354
GO:0003014~renal system process	2	0.027904137	ADORA2A, DRD2	66.60784314	0.9999998	0.200313685	33.79936354
GO:0019226~transmission of nerve impulse	3	0.028352027	ADORA2A, DRD2, DRD1A	10.61009891	0.999999844	0.200629265	34.24253408
GO:0051952~regulation of amine transport	2	0.02905081	ADORA2A, DRD2	63.94352941	0.999999895	0.202520317	34.92844162

		4					
GO:0007200~activation of phospholipase C activity by G-protein coupled receptor protein signaling pathway coupled to IP3 second messenger	2	0.02905081<br>4	DRD2, DRD1A	63.94352941	0.999999895	0.202520317	34.92844162
GO:0043005~neuron projection	3	0.02979634<br>2	CALCA, ADORA2A, DRD2	10.20734694	0.792570178	0.408042676	25.46424867
GO:0010863~positive regulation of phospholipase C activity	2	0.03019622<br>3	DRD2, DRD1A	61.4841629	0.999999945	0.207125773	36.038344
GO:0007202~activation of phospholipase C activity	2	0.03019622<br>3	DRD2, DRD1A	61.4841629	0.999999945	0.207125773	36.038344
GO:0010518~positive regulation of phospholipase activity	2	0.03019622<br>3	DRD2, DRD1A	61.4841629	0.999999945	0.207125773	36.038344
GO:0060193~positive regulation of lipase activity	2	0.03134036<br>6	DRD2, DRD1A	59.20697168	0.999999971	0.211580049	37.12939497
GO:0007628~adult walking behavior	2	0.03134036<br>6	DRD2, DRD1A	59.20697168	0.999999971	0.211580049	37.12939497
GO:0042311~vasodilation	2	0.03134036<br>6	CALCA, ADORA2A	59.20697168	0.999999971	0.211580049	37.12939497

GO:0007626~locomotory behavior	3	0.031435362	ADORA2A, DRD2, DRD1A	10.03298056	0.999999972	0.20961429	37.21919917
GO:0003073~regulation of systemic arterial blood pressure	2	0.032483243	CALCA, DRD2	57.09243697	0.999999985	0.213343622	38.20191336
GO:0010517~regulation of phospholipase activity	2	0.032483243	DRD2, DRD1A	57.09243697	0.999999985	0.213343622	38.20191336
GO:0008542~visual learning	2	0.033624856	DRD2, DRD1A	55.12373225	0.999999992	0.217508912	39.25621264
GO:0009628~response to abiotic stimulus	3	0.034395814	CALCA, DRD2, DRD1A	9.553316147	0.999999995	0.219434239	39.95870265
GO:0007632~visual behavior	2	0.035904293	DRD2, DRD1A	51.56736243	0.999999998	0.225458612	41.31138137
GO:0040013~negative regulation of locomotion	2	0.035904293	ADORA2A, DRD2	51.56736243	0.999999998	0.225458612	41.31138137
GO:0006873~cellular ion homeostasis	3	0.036944276	CALCA, ADORA2A, DRD1A	9.187288709	0.999999999	0.228713961	42.22736203
GO:0060191~regulation of lipase activity	2	0.037042121	DRD2, DRD1A	49.95588235	0.999999999	0.22674156	42.31285171
GO:0021954~central nervous system neuron	2	0.03817869	DRD2, DRD1A	48.44206774	0.999999999	0.230422413	43.29730487

development							
GO:0055082~cellular chemical homeostasis	3	0.038771183	CALCA, ADORA2A, DRD1A	8.947322212	1	0.231115812	43.80427365
GO:0050877~neurological system process	6	0.039006407	CALCA, GSX2, ADORA2A, DRD2, RGS9, DRD1A	2.85292368	1	0.229915873	44.00437033
GO:0032102~negative regulation of response to external stimulus	2	0.041580855	ADORA2A, DRD2	44.40522876	1	0.240845449	46.15141638
GO:0033555~multicellular organismal response to stress	2	0.042712401	CALCA, DRD1A	43.20508744	1	0.244127506	47.07063203
GO:0007267~cell-cell signaling	3	0.044735864	ADORA2A, DRD2, DRD1A	8.268559838	1	0.251764537	48.67806503
GO:0048015~phosphoinositide-mediated signaling	2	0.046099528	DRD2, DRD1A	39.96470588	1	0.255954228	49.73558115
GO:0021953~central nervous system neuron differentiation	2	0.048351367	DRD2, DRD1A	38.06162465	1	0.264298154	51.43755523

B. Enriched GO terms of down-regulated DEGs in long-term PFC.

Term	Count	PValue	Genes	Fold Enrichment	Bonferroni	Benjamini	FDR
GO:0005833~hemoglobin complex	4	1.02E-06	HBA-A2, HBB-BH1, HBB-B2, HBB-Y	178.6285714	9.68E-05	9.68E-05	0.00112027
GO:0015671~oxygen transport	4	4.45E-06	HBA-A2, HBB-BH1, HBB-B2, HBB-Y	114.9090909	0.001602047	0.001602047	0.006116548
GO:0005344~oxygen transporter activity	4	5.12E-06	HBA-A2, HBB-BH1, HBB-B2, HBB-Y	109.8181818	7.26E-04	7.26E-04	0.006050134
GO:0015669~gas transport	4	7.69E-06	HBA-A2, HBB-BH1, HBB-B2, HBB-Y	97.23076923	0.002763341	0.001382627	0.010556228
GO:0019825~oxygen binding	4	1.12E-05	HBA-A2, HBB-BH1, HBB-B2, HBB-Y	86.28571429	0.001590275	7.95E-04	0.013256383
GO:0044445~cytosolic part	4	6.53E-04	HBA-A2, HBB-BH1, HBB-B2, HBB-Y	22.6829932	0.060125798	0.030528906	0.714914919
GO:0020037~heme binding	4	0.011208585	HBA-A2, HBB-BH1, HBB-B2, HBB-Y	8.388888889	0.798225829	0.413472304	12.48217873
GO:0046906~tetrapyrrole binding	4	0.012736312	HBA-A2, HBB-BH1, HBB-B2, HBB-Y	8	0.838002155	0.365579352	14.06828514
GO:0043167~ion binding	21	0.014007601	GAL3ST3, CACNA2D1, HBB-BH1, CAR13, S100A8, S100A9, CLCNKA, ANXA1, BRSK1, ATMIN, HBB-Y, GCH1, RNF125, HBA-A2, UMODL1, TRIM9, CH25H, PLA2G12A, ADAMTS1, HBB-B2, KLF1	1.612099644	0.865089261	0.330101038	15.36804539
GO:0006955~immune response	6	0.014494923	RNF125, CXCL1, H2-Q10, CD8B1, SERPINA3G, PGLYRP1	4.025477707	0.994785751	0.826594084	18.17038699

GO:0005829~cytosol	6	0.015537843	HBA-A2, HBB-BH1, IRS2, RAPGEF4, HBB-B2, HBB-Y	3.904449649	0.774104527	0.390973988	15.81332177
GO:0046872~metal ion binding	20	0.023904158	GAL3ST3, CACNA2D1, HBB-BH1, CAR13, S100A8, S100A9, ANXA1, BRSK1, ATMIN, HBB-Y, GCH1, RNF125, HBA-A2, UMODL1, TRIM9, CH25H, PLA2G12A, ADAMTS1, HBB-B2, KLF1	1.568831169	0.967794527	0.435944792	24.88734588
GO:0005506~iron ion binding	5	0.024414559	HBA-A2, HBB-BH1, CH25H, HBB-B2, HBB-Y	4.402332362	0.9700998	0.394325687	25.3506084
GO:0043169~cation binding	20	0.026232398	GAL3ST3, CACNA2D1, HBB-BH1, CAR13, S100A8, S100A9, ANXA1, BRSK1, ATMIN, HBB-Y, GCH1, RNF125, HBA-A2, UMODL1, TRIM9, CH25H, PLA2G12A, ADAMTS1, HBB-B2, KLF1	1.554697555	0.977056714	0.376147452	26.97939404
GO:0046903~secretion	4	0.030606673	TRIM9, CLCNKA, ANXA1, RAPGEF4	5.719457014	0.999986196	0.939045981	34.74848849
GO:0045120~pronucleus	2	0.047861142	HNRNPL, TAF1	39.6952381	0.990526128	0.688016315	41.67260733
GO:0048821~erythrocyte development	2	0.048351367	HBA-A2, KLF1	39.5	0.999999982	0.971795576	49.37130112

C. Enriched GO terms of up-regulated DEGs in long-term hippocampus.

Term	Count	PValue	Genes	Fold Enrichment	Bonferroni	Benjamini	FDR
GO:0005576~extracellular region	32	3.99E-06	LEPR, ELN, 1500015O10RIK, IL17RE, TRH, SCT, LGALS3BP, CTGF, SOSTDC1, EMID2, LEFTY2, TFF3, SEMA3A, LBP, WNT6, DEFB2, DKKL1, COL8A2, FGFBP1, BMP3, CPA6, KL, IL25, IGF2, CBLN1, SFRP1, TFPI, IGFBP2, MUP8, WFDC2, PON3, BMP6	2.358132956	5.23E-04	5.23E-04	0.00465494
GO:0044421~extracellular region part	17	4.01E-04	BMP3, LEPR, ELN, IGF2, LGALS3BP, CTGF, SOSTDC1, EMID2, LEFTY2, TFF3, LBP, WNT6, IGFBP2, COL8A2, FGFBP1, PON3, BMP6	2.71916494	0.051152986	0.025912214	0.466323116
GO:0005344~oxygen transporter activity	3	0.002892738	HBB-BH1, NGB, HBB-Y	36.24	0.519497674	0.519497674	3.700254518
GO:0015671~oxygen transport	3	0.003225748	HBB-BH1, NGB, HBB-Y	34.31313131	0.974367939	0.974367939	5.05440823
GO:0051216~cartilage development	5	0.003316221	MEF2C, BMP3, MSX1, CTGF, BMP6	8.065052232	0.976875056	0.847931122	5.192652979
GO:0005506~iron ion binding	9	0.004051161	1600014K23RIK, HBB-BH1, CYP2C44, CYP4F14, GUCY1B2, NGB, ASPH, SCARA5, HBB-Y	3.48664723	0.641929489	0.401610067	5.146283199

GO:0020037~heme binding	6	0.004412109	HBB-BH1, CYP2C44, CYP4F14, GUCY1B2, NGB, HBB-Y	5.536666667	0.673306645	0.311273542	5.592732447
GO:0015669~gas transport	3	0.004527944	HBB-BH1, NGB, HBB-Y	29.03418803	0.994179378	0.820117131	7.026110527
GO:0019825~oxygen binding	3	0.004717005	HBB-BH1, NGB, HBB-Y	28.47428571	0.697666886	0.258482458	5.968340411
GO:0046906~tetrapyrrole binding	6	0.005388669	HBB-BH1, CYP2C44, CYP4F14, GUCY1B2, NGB, HBB-Y	5.28	0.745134647	0.239214617	6.790914753
GO:0001503~ossification	5	0.009770853	MEF2C, BMP3, CTGF, IGF2, BMP6	5.934661076	0.999985402	0.938188046	14.58269043
GO:0043176~amine binding	4	0.010565074	FOLR2, FOLR1, HTR1D, DRD1A	8.713442623	0.931927633	0.36100841	12.91099295
GO:0009986~cell surface	8	0.011128009	KLRC2, COL23A1, SLC6A2, H2-Q1, VPREB1, CLEC2D, SCARA5, FGFBP1	3.247265054	0.769139315	0.38654413	12.23394077
GO:0009891~positive regulation of biosynthetic process	11	0.012341463	MEF2C, HSP90AB1, BMP3, TCF21, MSX1, HES5, ADORA2A, NLRP12, CENPK, KAT5, CITED4	2.484673183	0.999999234	0.940182643	18.07355989
GO:0060348~bone development	5	0.014054577	MEF2C, BMP3, CTGF, IGF2, BMP6	5.331136221	0.999999893	0.931104099	20.32518162
GO:0009628~response to abiotic stimulus	7	0.014364863	ARPP21, BRCA2, TRPV4, PCDH15, DRD1A, SCARA5, SLC14A2	3.508779696	0.999999925	0.904055351	20.72674403
GO:0005886~plasma membrane	34	0.016359916	KLRC2, CLDN9, ECEL1, ADORA2A, SLC6A2, TACR1, LEPR, PRSS41, F2RL1, VPREB1, AQP1,	1.448474648	0.884775567	0.417378986	17.49876631

			GPR88, VMN1R208, FOLR2, FOLR1, KRT8, CAMK2D, CLEC2D, TRPV4, HTR1D, FGFBP1, COL23A1, CNTN5, KL, AK1, H2-Q1, PCDH15, CBLN1, GABRR1, VMN1R202, OTOF, PERP, DRD1A, SCARA5				
GO:0019838~growth factor binding	4	0.016511706	KL, CTGF, IGFBP2, FGFBP1	7.382222222	0.98518825	0.452154559	19.48280213
GO:0010033~response to organic substance	10	0.018084406	IRAK1, MSX1, ADH1, ADORA2A, LEPR, VMN1R202, LBP, TRH, DRD1A, VMN1R208	2.491382472	0.999999999	0.924749085	25.39506111
GO:0007628~adult walking behavior	3	0.018968627	PCDH15, TRH, DRD1A	13.97942387	1	0.910454266	26.46624917
GO:0048608~reproductive structure development	5	0.019375831	TCF21, COL9A3, SFRP1, LEPR, BRCA2	4.839031339	1	0.891258825	26.95468997
GO:0005615~extracellular space	10	0.020999042	BMP3, LEPR, SOSTDC1, LEFTY2, IGF2, LBP, IGFBP2, FGFBP1, PON3, BMP6	2.422739339	0.937971926	0.426520814	21.92383832
GO:0008083~growth factor activity	5	0.021058753	BMP3, CTGF, LEFTY2, IGF2, BMP6	4.712056738	0.995414077	0.489872954	24.19560034
GO:0051173~positive regulation of nitrogen	10	0.022863155	MEF2C, HSP90AB1, BMP3, TCF21, MSX1, HES5, ADORA2A, CENPK, KAT5, CITED4	2.391916631	1	0.907850711	31.01489868

compound metabolic process							
GO:0043279~response to alkaloid	3	0.023152265	ADORA2A, LEPR, DRD1A	12.58148148	1	0.890695111	31.34182393
GO:0006620~posttranslational protein targeting to membrane	2	0.023439958	FOLR2, FOLR1	83.87654321	1	0.873691809	31.66570425
GO:0006978~DNA damage response, signal transduction by p53 class mediator resulting in transcription of p21 class mediator	2	0.023439958	BRCA2, KAT5	83.87654321	1	0.873691809	31.66570425
GO:0051968~positive regulation of synaptic transmission, glutamatergic	2	0.023439958	ADORA2A, DRD1A	83.87654321	1	0.873691809	31.66570425
GO:0042772~DNA damage response, signal transduction resulting in transcription	2	0.023439958	BRCA2, KAT5	83.87654321	1	0.873691809	31.66570425
GO:0006952~defense response	9	0.02503815	IRAK1, H2-Q1, IL25, CLEC2D, C1S, LBP, DEFB2, DRD1A, BMP6	2.527529762	1	0.871767712	33.43899478
GO:0031328~positive	10	0.029964084	MEF2C, HSP90AB1, BMP3, TCF21, MSX1, HES5,	2.279253892	1	0.899732829	38.63698755

regulation of cellular biosynthetic process			ADORA2A, CENPK, KAT5, CITED4				
GO:0005578~proteinaceous extracellular matrix	7	0.031766344	LGALS3BP, CTGF, ELN, EMID2, TFF3, WNT6, COL8A2	2.917891789	0.9854319	0.505802569	31.37070461
GO:0033555~multicellular organismal response to stress	3	0.0342113	SLC6A2, TACR1, DRD1A	10.2012012	1	0.915175711	42.81069386
GO:0008517~folic acid transporter activity	2	0.036706144	FOLR2, FOLR1	53.152	0.999922201	0.650504533	38.53679334
GO:0030534~adult behavior	4	0.036987651	ADH1, PCDH15, TRH, DRD1A	5.411389885	1	0.919062734	45.39347642
GO:0031012~extracellular matrix	7	0.037440215	LGALS3BP, CTGF, ELN, EMID2, TFF3, WNT6, COL8A2	2.804575603	0.993254247	0.510377359	35.91678006
GO:0032535~regulation of cellular component size	5	0.038316014	ELN, LEFTY2, VPREB1, SEMA3A, VILL	3.907292386	1	0.914682093	46.59015882
GO:0007626~locomotory behavior	6	0.040486518	ADORA2A, PCDH15, SEMA3A, LBP, TRH, DRD1A	3.158530916	1	0.915134151	48.49272493
GO:0007610~behavior	8	0.040770218	ADH1, ADORA2A, LEPR, PCDH15, SEMA3A, LBP, TRH, DRD1A	2.48523091	1	0.905590764	48.73665393
GO:0014070~response to organic cyclic substance	3	0.043125482	ADORA2A, LEPR, DRD1A	8.986772487	1	0.9074941	50.72031945

GO:0014002~astrocyte development	2	0.046335499	ADORA2A, DRD1A	41.9382716	1	0.913316249	53.30818228
GO:0045935~positive regulation of nucleobase, nucleoside, nucleotide and nucleic acid metabolic process	9	0.047984744	MEF2C, BMP3, TCF21, MSX1, HES5, ADORA2A, CENPK, KAT5, CITED4	2.220261438	1	0.911477878	54.5876778

D. Enriched GO terms of down-regulated DEGs in long-term hippocampus.

Term	Count	PValue	Genes	Fold Enrichment	Bonferroni	Benjamini	FDR
GO:0019911~structural constituent of myelin sheath	3	9.15E-05	PLP1, MOBP, MBP	177.1733333	0.015067898	0.015067898	0.111070841
GO:0043209~myelin sheath	3	8.46E-04	PLP1, ERMN, MBP	66.04225352	0.093556146	0.093556146	0.961422394
GO:0042552~myelination	4	0.001139563	PLP1, UGT8A, NKX6-2, MBP	19.07757108	0.686029392	0.686029392	1.788952175
GO:0051094~positive regulation of developmental process	7	0.001243182	FGFR2, EPC1, WNT7B, LECT1, NKX6-2, INPP5D, FOXP3	5.772302464	0.717438336	0.468434704	1.950123621
GO:0008366~axon ensheathment	4	0.001329641	PLP1, UGT8A, NKX6-2, MBP	18.0992341	0.741229672	0.362757364	2.084414842
GO:0007272~ensheathment of neurons	4	0.001329641	PLP1, UGT8A, NKX6-2, MBP	18.0992341	0.741229672	0.362757364	2.084414842
GO:0019228~regulation of action potential in neuron	4	0.002015502	PLP1, UGT8A, NKX6-2, MBP	15.68600289	0.871241503	0.400976343	3.14362518
GO:0006917~induction of apoptosis	6	0.002415794	PLEKHF1, PTPN6, MAL, TRAF6, MYC, GCH1	6.340150867	0.914344059	0.388281665	3.756846199
GO:0012502~induction of programmed cell death	6	0.002415794	PLEKHF1, PTPN6, MAL, TRAF6, MYC, GCH1	6.340150867	0.914344059	0.388281665	3.756846199
GO:0043065~positive regulation of apoptosis	7	0.002619506	PLEKHF1, PTPN6, MAL, INPP5D, TRAF6, MYC, GCH1	4.980938416	0.930394413	0.358631907	4.067521052
GO:0043068~positive regulation of	7	0.002726187	PLEKHF1, PTPN6, MAL, INPP5D,	4.941090909	0.937562458	0.327146016	4.229841506

programmed cell death			TRAF6, MYC, GCH1				
GO:0010942~positive regulation of cell death	7	0.002836104	PLEKHF1, PTPN6, MAL, INPP5D, TRAF6, MYC, GCH1	4.901875902	0.944177333	0.302809721	4.396816326
GO:0001508~regulation of action potential	4	0.003398575	PLP1, UGT8A, NKX6-2, MBP	13.07166907	0.968533553	0.319082981	5.247009335
GO:0042981~regulation of apoptosis	10	0.003607006	PLEKHF1, PTPN6, MITF, HSPA1A, MAL, HSPA1B, INPP5D, TRAF6, MYC, TIMP1, GCH1	3.19109462	0.974557736	0.307283432	5.560256221
GO:0043067~regulation of programmed cell death	10	0.003918084	PLEKHF1, PTPN6, MITF, HSPA1A, MAL, HSPA1B, INPP5D, TRAF6, MYC, TIMP1, GCH1	3.151205937	0.981474225	0.304134788	6.025965396
GO:0010941~regulation of cell death	10	0.004057706	PLEKHF1, PTPN6, MITF, HSPA1A, MAL, HSPA1B, INPP5D, TRAF6, MYC, TIMP1, GCH1	3.134414431	0.983933458	0.291249245	6.234290913
GO:0006955~immune response	9	0.004699487	CXCL1, PTPN6, CCL3, CXCL5, H2-M2, INPP5D, CLEC7A, TRAF6, FOXP3	3.371991066	0.991653673	0.307985764	7.18631872
GO:0048709~oligodendrocyte differentiation	3	0.005495524	SOX10, PLP1, NKX6-2	26.47012987	0.996297885	0.329624942	8.354590237

GO:0044092~negative regulation of molecular function	5	0.006365479	OPRM1, PTPN6, HSPA1A, HSPA1B, FOXP3, MYC	6.68437623	0.998478408	0.3511367	9.615582497
GO:0050777~negative regulation of immune response	3	0.009198954	PTPN6, INPP5D, FOXP3	20.36163836	0.999916394	0.443916529	13.6109131
GO:0007626~locomotory behavior	6	0.010839363	OPRM1, CCL3, CXCL5, S100A8, S100A9, CNP	4.430147259	0.999984473	0.478657374	15.84771274
GO:0043565~sequence-specific DNA binding	9	0.011910471	MAFF, SOX10, RHOX3A, NKX6-2, HOXC5, MITF, EN2, FOXP3, MYC	2.867913669	0.863168755	0.630092923	13.54881356
GO:0003705~RNA polymerase II transcription factor activity, enhancer binding	3	0.012033263	SOX10, MITF, FOXP3	17.71733333	0.865962716	0.488229549	13.67926423
GO:0032675~regulation of interleukin-6 production	3	0.013739145	INPP5D, TRAF6, FOXP3	16.54383117	0.999999214	0.541994937	19.66957366
GO:0032880~regulation of protein localization	4	0.013949649	SGK1, RABGAP1L, TRAF6, FOXP3	7.843001443	0.999999367	0.528195606	19.94058533
GO:0042592~homeostatic process	9	0.016070021	SGK1, PLP1, UGT8A, NKX6-2, IKBKG, HSPA1A, HSPA1B, FOXP3, TIMP1, MBP	2.719533891	0.999999929	0.560882222	22.62308763
GO:0050672~negative regulation of	3	0.016304793	PTPN6, INPP5D, FOXP3	15.1257885	0.999999944	0.548573684	22.91486543

lymphocyte proliferation							
GO:0070664~negative regulation of leukocyte proliferation	3	0.016304793	PTPN6, INPP5D, FOXP3	15.1257885	0.999999944	0.548573684	22.91486543
GO:0032945~negative regulation of mononuclear cell proliferation	3	0.016304793	PTPN6, INPP5D, FOXP3	15.1257885	0.999999944	0.548573684	22.91486543
GO:0045597~positive regulation of cell differentiation	5	0.016627626	EPC1, WNT7B, NKX6-2, INPP5D, FOXP3	5.041929499	0.99999996	0.538996559	23.3144034
GO:0033269~internode region of axon	2	0.01670211	ERMN, MBP	117.4084507	0.858266518	0.623524925	17.48257915
GO:0008009~chemokine activity	3	0.018898819	CXCL1, CCL3, CXCL5	13.98736842	0.95787979	0.546974517	20.69214525
GO:0042379~chemokine receptor binding	3	0.019849456	CXCL1, CCL3, CXCL5	13.62871795	0.964140657	0.48605048	21.62084744
GO:0001501~skeletal system development	6	0.021618812	FGFR2, LECT1, HOXC5, TRAF6, MOG, MYC	3.715105947	1	0.619192614	29.24987238
GO:0002429~immune response-activating cell surface receptor signaling pathway	3	0.021992292	PTPN6, CLEC7A, FOXP3	12.91225847	1	0.609917535	29.67624013
GO:0022010~myelination in the central nervous system	2	0.022188108	PLP1, NKX6-2	88.23376623	1	0.598231404	29.89882307
GO:0032291~ensheathment of axons in	2	0.022188108	PLP1, NKX6-2	88.23376623	1	0.598231404	29.89882307

the central nervous system							
GO:0010605~negative regulation of macromolecule metabolic process	8	0.022817542	EPC1, PTPN6, RHOX3A, NKX6-2, ZFP639, INPP5D, FOXP3, MYC	2.790000513	1	0.594230203	30.60983168
GO:0042330~taxis	4	0.023125139	CCL3, CXCL5, S100A8, S100A9	6.475872751	1	0.585387084	30.95483103
GO:0006935~chemotaxis	4	0.023125139	CCL3, CXCL5, S100A8, S100A9	6.475872751	1	0.585387084	30.95483103
GO:0002768~immune response-regulating cell surface receptor signaling pathway	3	0.0251007	PTPN6, CLEC7A, FOXP3	12.03187721	1	0.60244524	33.13259203
GO:0003700~transcription factor activity	10	0.027844318	MAFF, SOX10, RHOX3A, NKX6-2, HOXC5, MITF, ZFP639, EN2, FOXP3, MYC	2.283161512	0.990792411	0.54218398	29.04538389
GO:0045892~negative regulation of transcription, DNA-dependent	6	0.028972384	EPC1, RHOX3A, NKX6-2, ZFP639, FOXP3, MYC	3.437679204	1	0.643002599	37.21519556
GO:0042391~regulation of membrane potential	4	0.028998166	PLP1, UGT8A, NKX6-2, MBP	5.931681764	1	0.630864601	37.24158207
GO:0010558~negative regulation of macromolecule biosynthetic process	7	0.029344063	EPC1, RHOX3A, NKX6-2, ZFP639, INPP5D, FOXP3, MYC	2.955197912	1	0.623230152	37.5945848
GO:0002757~immune response-activating signal transduction	3	0.029505104	PTPN6, CLEC7A, FOXP3	11.02922078	1	0.613601108	37.75829874

GO:0010001~glial cell differentiation	3	0.029505104	SOX10, PLP1, NKX6-2	11.02922078	1	0.613601108	37.75829874
GO:0051253~negative regulation of RNA metabolic process	6	0.029678251	EPC1, RHOX3A, NKX6-2, ZFP639, FOXP3, MYC	3.415500628	1	0.604483881	37.93387095
GO:0031327~negative regulation of cellular biosynthetic process	7	0.03304789	EPC1, RHOX3A, NKX6-2, ZFP639, INPP5D, FOXP3, MYC	2.872727273	1	0.63367545	41.25970041
GO:0002764~immune response-regulating signal transduction	3	0.034192871	PTPN6, CLEC7A, FOXP3	10.18081918	1	0.635757935	42.35125793
GO:0051250~negative regulation of lymphocyte activation	3	0.034192871	PTPN6, INPP5D, FOXP3	10.18081918	1	0.635757935	42.35125793
GO:0042063~gliogenesis	3	0.034192871	SOX10, PLP1, NKX6-2	10.18081918	1	0.635757935	42.35125793
GO:0009890~negative regulation of biosynthetic process	7	0.034347445	EPC1, RHOX3A, NKX6-2, ZFP639, INPP5D, FOXP3, MYC	2.846250524	1	0.627083249	42.49715594
GO:0030247~polysaccharide binding	4	0.034498539	FGFR2, TNFAIP6, LIPG, CLEC7A	5.536666667	0.997055665	0.565062603	34.72659028
GO:0001871~pattern binding	4	0.034498539	FGFR2, TNFAIP6, LIPG, CLEC7A	5.536666667	0.997055665	0.565062603	34.72659028
GO:0002695~negative regulation of leukocyte activation	3	0.035407391	PTPN6, INPP5D, FOXP3	9.988728253	1	0.628385387	43.48832353
GO:0050866~negative regulation of cell activation	3	0.035407391	PTPN6, INPP5D, FOXP3	9.988728253	1	0.628385387	43.48832353
GO:0050864~regulation of B cell	3	0.037885977	PTPN6, INPP5D, FOXP3	9.625501771	1	0.643933784	45.74395833

activation							
GO:0043473~pigmentation	3	0.039149609	SOX10, MITF, MYC	9.453617811	1	0.646686201	46.8611923
GO:0000122~negative regulation of transcription from RNA polymerase II promoter	5	0.040390134	EPC1, RHOX3A, NKX6-2, FOXP3, MYC	3.81964356	1	0.649082121	47.93700075
GO:0045619~regulation of lymphocyte differentiation	3	0.041724479	PTPN6, INPP5D, FOXP3	9.12763099	1	0.652205314	49.07137621
GO:0001817~regulation of cytokine production	4	0.042885669	INPP5D, CLEC7A, TRAF6, FOXP3	5.078202373	1	0.653657553	50.03966134
GO:0045165~cell fate commitment	4	0.049222374	FGFR2, WNT3, NKX6-2, MITF	4.801837618	1	0.696574872	55.02687019
GO:0042035~regulation of cytokine biosynthetic process	3	0.049814847	INPP5D, TRAF6, FOXP3	8.271915584	1	0.692693259	55.46850558

E. Enriched GO terms of up-regulated DEGs in short-term PFC.

Term	Count	PValue	Genes	Fold Enrichment	Bonferroni	Benjamini	FDR
GO:0030017~sarcomere	10	7.18E-08	TNNT2, ACTC1, DES, TCAP, PDLIM5, LDB3, MYOZ2, TNNI3, TPM1, CSRP3	12.81147541	1.41E-05	1.41E-05	8.98E-05
GO:0044449~contractile fiber part	10	1.36E-07	TNNT2, ACTC1, DES, TCAP, PDLIM5, LDB3, MYOZ2, TNNI3, TPM1, CSRP3	11.91765154	2.67E-05	1.34E-05	1.70E-04
GO:0030016~myofibril	10	2.22E-07	TNNT2, ACTC1, DES, TCAP, PDLIM5, LDB3, MYOZ2, TNNI3, TPM1, CSRP3	11.26283553	4.37E-05	1.46E-05	2.78E-04
GO:0043292~contractile fiber	10	3.22E-07	TNNT2, ACTC1, DES, TCAP, PDLIM5, LDB3, MYOZ2, TNNI3, TPM1, CSRP3	10.78861087	6.35E-05	1.59E-05	4.03E-04
GO:0048738~cardiac muscle tissue development	8	8.97E-07	TNNT2, ACTC1, XIRP1, GATA6, PLN, NKX2-5, TPM1, CSRP3	15.01643873	9.45E-04	9.45E-04	0.001426997
GO:0007517~muscle organ development	11	5.43E-06	TNNT2, APP, ACTC1, DES, XIRP1, GATA6, PLN, ETV1, NKX2-5, TPM1, CSRP3	6.687007874	0.005705993	0.002857078	0.008635034
GO:0031674~I band	7	1.15E-05	ACTC1, DES, TCAP, PDLIM5, LDB3, MYOZ2, CSRP3	13.53665326	0.002259003	4.52E-04	0.014352334
GO:0014706~striated muscle tissue development	9	2.33E-05	TNNT2, APP, ACTC1, XIRP1, GATA6, PLN, NKX2-5, TPM1, CSRP3	7.582119164	0.024267717	0.008155572	0.037066514

GO:0060537~muscle tissue development	9	3.82E-05	TNNT2, APP, ACTC1, XIRP1, GATA6, PLN, NKX2-5, TPM1, CSRP3	7.080361278	0.039442316	0.010009875	0.060708444
GO:0007507~heart development	11	4.25E-05	TNNT2, ACTC1, TCAP, XIRP1, GATA6, PLN, NPPB, TNNI3, NKX2-5, TPM1, CSRP3	5.277638501	0.043758864	0.008909109	0.067500821
GO:0030018~Z disc	6	7.80E-05	DES, TCAP, PDLIM5, LDB3, MYOZ2, CSRP3	13.36849608	0.015240275	0.002556327	0.097422152
GO:0008016~regulation of heart contraction	6	9.60E-05	TNNT2, GNAI3, PLN, NKX2-5, TPM1, CSRP3	12.83905512	0.096280141	0.016731097	0.152655424
GO:0051146~striated muscle cell differentiation	7	1.75E-04	TNNT2, APP, ACTC1, TCAP, XIRP1, GATA6, NKX2-5	8.415111032	0.168327959	0.025987376	0.277761053
GO:0055001~muscle cell development	6	2.49E-04	TNNT2, APP, ACTC1, TCAP, XIRP1, NKX2-5	10.52381567	0.230549063	0.032228972	0.394713303
GO:0044057~regulation of system process	9	5.64E-04	TNNT2, GNAI3, TNNC1, PLN, TNNI3, NKX2-5, TPM1, CSRP3, CASQ2	4.790692208	0.44846247	0.063977865	0.893945871
GO:0030239~myofibril assembly	4	6.77E-04	TNNT2, ACTC1, TCAP, XIRP1	22.5246581	0.510109817	0.068870956	1.071056532
GO:0042692~muscle cell differentiation	7	7.62E-04	TNNT2, APP, ACTC1, TCAP, XIRP1, GATA6, NKX2-5	6.401238307	0.552399668	0.070471385	1.20574526
GO:0031032~actomyosin structure organization	4	0.00154253	TNNT2, ACTC1, TCAP, XIRP1	17.11874016	0.803497905	0.126799588	2.425482695
GO:0055002~striated muscle cell	5	0.00155807	TNNT2, APP, ACTC1, TCAP, XIRP1	9.906678332	0.806695011	0.118757122	2.449633929

development							
GO:0055007~cardiac muscle cell differentiation	4	0.002153057	ACTC1, XIRP1, GATA6, NKX2-5	15.28458943	0.896870333	0.149787666	3.370159736
GO:0044093~positive regulation of molecular function	10	0.002181731	MEN1, OPRM1, ADCY3, CASP8AP2, IKBKG, PICK1, NRK, LPAR1, APAF1, NKX2-5	3.496474705	0.899947145	0.142273886	3.414316717
GO:0005200~structural constituent of cytoskeleton	4	0.002715894	TNNT2, DES, ODF2, TPM1	14.09867374	0.489180189	0.489180189	3.464845727
GO:0043085~positive regulation of catalytic activity	9	0.002975534	MEN1, OPRM1, ADCY3, CASP8AP2, IKBKG, PICK1, NRK, LPAR1, APAF1	3.689383655	0.956755634	0.178238868	4.629246745
GO:0035051~cardiac cell differentiation	4	0.003466997	ACTC1, XIRP1, GATA6, NKX2-5	12.96874254	0.974282009	0.193722516	5.374245865
GO:0016563~transcription activator activity	9	0.003752416	CUTA, NACA, GATA6, MITF, ETV1, NPAS4, FOXP3, NKX2-5, HMGA1	3.551885952	0.604889577	0.371421904	4.757736133
GO:0045214~sarcomere organization	3	0.004441982	TNNT2, TCAP, XIRP1	29.17967072	0.990834217	0.229474175	6.836091743
GO:0010927~cellular component assembly involved in morphogenesis	4	0.004446445	TNNT2, ACTC1, TCAP, XIRP1	11.888014	0.990877423	0.21902386	6.842734407
GO:0003007~heart	5	0.004918332	TNNT2, ACTC1, XIRP1, NKX2-5, TPM1	7.229197702	0.994465265	0.22882163	7.542587331

morphogenesis							
GO:0006937~regulation of muscle contraction	4	0.006419329	TNNC1, TNNI3, NKX2-5, CASQ2	10.43825619	0.998872534	0.276190997	9.73608623
GO:0055006~cardiac cell development	3	0.007217066	ACTC1, XIRP1, NKX2-5	22.92688414	0.999516457	0.293206536	10.88192649
GO:0055013~cardiac muscle cell development	3	0.007217066	ACTC1, XIRP1, NKX2-5	22.92688414	0.999516457	0.293206536	10.88192649
GO:0045859~regulation of protein kinase activity	7	0.007715875	CAV3, APP, IKBKG, PICK1, FABP4, NRK, LPAR1	4.026585387	0.999715299	0.298798191	11.59145678
GO:0043549~regulation of kinase activity	7	0.008950853	CAV3, APP, IKBKG, PICK1, FABP4, NRK, LPAR1	3.900754593	0.999923382	0.32622662	13.32543569
GO:0051338~regulation of transferase activity	7	0.010563808	CAV3, APP, IKBKG, PICK1, FABP4, NRK, LPAR1	3.76354212	0.999986236	0.360928706	15.54214596
GO:0055008~cardiac muscle tissue morphogenesis	3	0.011844349	TNNT2, ACTC1, TPM1	17.832021	0.999996485	0.383081197	17.26406057
GO:0060415~muscle tissue morphogenesis	3	0.011844349	TNNT2, ACTC1, TPM1	17.832021	0.999996485	0.383081197	17.26406057
GO:0010558~negative regulation of macromolecule biosynthetic	10	0.015777482	MEN1, SATB1, RHOX3A, EIF4EBP2, TIMELESS, NR6A1, FABP4, ZFP639, FOXP3,	2.559620239	0.999999947	0.462494663	22.34941843

process			NKX2-5				
GO:0043009~chordate embryonic development	10	0.016454675	MEN1, WNT7B, TCAP, GATA6, NASP, DAD1, APAF1, TRAF6, NKX2-5, TPM1	2.541380665	0.999999975	0.464499572	23.1948691
GO:0009792~embryonic development ending in birth or egg hatching	10	0.017390587	MEN1, WNT7B, TCAP, GATA6, NASP, DAD1, APAF1, TRAF6, NKX2-5, TPM1	2.517461788	0.999999991	0.471450274	24.34913025
GO:0031327~negative regulation of cellular biosynthetic process	10	0.018614697	MEN1, SATB1, RHOX3A, EIF4EBP2, TIMELESS, NR6A1, FABP4, ZFP639, FOXP3, NKX2-5	2.488188976	0.999999997	0.483231325	25.83429944
GO:0009890~negative regulation of biosynthetic process	10	0.01963839	MEN1, SATB1, RHOX3A, EIF4EBP2, TIMELESS, NR6A1, FABP4, ZFP639, FOXP3, NKX2-5	2.465256359	0.999999999	0.490511858	27.05528421
GO:0000122~negative regulation of transcription from RNA polymerase II promoter	7	0.020612585	MEN1, SATB1, RHOX3A, TIMELESS, NR6A1, FOXP3, NKX2-5	3.242185636	1	0.496424636	28.19971678
GO:0002637~regulation of immunoglobulin production	3	0.022265957	TNFSF13, TRAF6, FOXP3	12.83905512	1	0.51285723	30.10358437
GO:0005856~cytoskeleton	19	0.022367384	ACTC1, MYL3, PICK1, CETN4, MICAL3, LDB3, MYOZ2, HOMER2, TPM1, CSRP3,	1.735600947	0.988395956	0.470926973	24.63544593

			TNNT2, APP, FRMD6, DES, MFAP2, ODF2, SKA1, CDC42EP4, SGCA				
GO:0016481~negative regulation of transcription	9	0.022485231	MEN1, SATB1, RHOX3A, TIMELESS, NR6A1, FABP4, ZFP639, FOXP3, NKX2-5	2.588519177	1	0.505890544	30.35249593
GO:0048729~tissue morphogenesis	7	0.023460626	TNNT2, ACTC1, TIMELESS, GRSF1, APAF1, TRAF6, TPM1	3.146827235	1	0.510768453	31.44969676
GO:0045597~positive regulation of cell differentiation	6	0.023467025	MEN1, WNT7B, CD36, GATA6, FOXP3, NKX2-5	3.668301462	1	0.501051489	31.45684124
GO:0045892~negative regulation of transcription, DNA-dependent	8	0.024608079	MEN1, SATB1, RHOX3A, TIMELESS, NR6A1, ZFP639, FOXP3, NKX2-5	2.779016259	1	0.508241231	32.71970321
GO:0051253~negative regulation of RNA metabolic process	8	0.025377589	MEN1, SATB1, RHOX3A, TIMELESS, NR6A1, ZFP639, FOXP3, NKX2-5	2.761087122	1	0.509818626	33.55901155
GO:0006357~regulation of transcription from RNA polymerase II promoter	12	0.027929135	MEN1, SATB1, APP, RHOX3A, TIMELESS, MED15, GATA6, MITF, NR6A1, NPAS4, FOXP3, NKX2-5	2.084262194	1	0.53491902	36.27232387
GO:0002700~regulation of production of molecular mediator of immune response	3	0.031332621	TNFSF13, TRAF6, FOXP3	10.6992126	1	0.567782171	39.73018756
GO:0045934~negative regulation	9	0.03146725	MEN1, SATB1, RHOX3A, TIMELESS, NR6A1,	2.425514191	1	0.560421767	39.86328346

of nucleobase, nucleoside, nucleotide and nucleic acid metabolic process			FABP4, ZFP639, FOXP3, NKX2-5				
GO:0005788~endoplasmic reticulum lumen	4	0.032527044	P4HA1, SDF2L1, HSPA5, CASQ2	5.693989071	0.998517993	0.557048387	33.86319777
GO:0051172~negative regulation of nitrogen compound metabolic process	9	0.03310425	MEN1, SATB1, RHOX3A, TIMELESS, NR6A1, FABP4, ZFP639, FOXP3, NKX2-5	2.401319536	1	0.570365778	41.45975825
GO:0003705~RNA polymerase II transcription factor activity, enhancer binding	3	0.034076709	GATA6, MITF, FOXP3	10.22153846	0.999809089	0.942419262	36.20861829
GO:0010629~negative regulation of gene expression	9	0.036998681	MEN1, SATB1, RHOX3A, TIMELESS, NR6A1, FABP4, ZFP639, FOXP3, NKX2-5	2.348607644	1	0.603111766	45.09958125
GO:0051924~regulation of calcium ion transport	3	0.039454414	CAV3, PLN, NKX2-5	9.440481704	1	0.618735676	47.28455128
GO:0030528~transcription regulator activity	19	0.043370971	CUTA, SATB1, NACA, NR6A1, MITF, ZFP639, NPAS4, FOXP3, HMGA1, MEN1, MAX, RHOX3A, MED15, CASP8AP2, GATA6, XBP1, ETV1, FABP4, NKX2-5	1.610358464	0.999982475	0.935298469	43.72499803

GO:0043280~positive regulation of caspase activity	3	0.04378222	MEN1, CASP8AP2, APAF1	8.916010499	1	0.649573138	50.93809842
GO:0010952~positive regulation of peptidase activity	3	0.04378222	MEN1, CASP8AP2, APAF1	8.916010499	1	0.649573138	50.93809842
GO:0042592~homeostatic process	11	0.044888145	MEN1, CAV3, APP, XIRP1, PLN, IKBKG, FABP4, TNNI3, FOXP3, CSRP3, GLRX2	2.015262647	1	0.650874287	51.83289363
GO:0051890~regulation of cardioblast differentiation	2	0.045519087	GATA6, NKX2-5	42.79685039	1	0.648221111	52.33650958
GO:0051891~positive regulation of cardioblast differentiation	2	0.045519087	GATA6, NKX2-5	42.79685039	1	0.648221111	52.33650958
GO:0010605~negative regulation of macromolecule metabolic process	10	0.045706679	MEN1, SATB1, RHOX3A, EIF4EBP2, TIMELESS, NR6A1, FABP4, ZFP639, FOXP3, NKX2-5	2.114468893	1	0.642028838	52.4852902
GO:0045935~positive regulation of nucleobase, nucleoside, nucleotide and nucleic acid metabolic process	10	0.047622527	MEN1, APP, GATA6, MITF, ETV1, TNFSF13, NPAS4, FOXP3, NKX2-5, HMGA1	2.097884823	1	0.649909987	53.98004961
GO:0007166~cell surface receptor linked signal transduction	32	0.048054537	OLFR1257, OPRM1, GNAI3, VMN1R185, OLFR156, TRHR, MITF, OLFR1502, OLFR638,	1.3722437	1	0.645886566	54.31096198

			LPAR1, OLFR434, GPR142, APP, EIF4EBP2, OLFR554, OLFR1462, TAS2R140, OLFR1183, OLFR699, RXFP4, VMN1R116, LTK, OLFR1280, PICK1, FOXP3, HOMER2, OLFR1431, WNT7B, OLFR644, WNT9B, OLFR1417, OLFR494				
GO:0051094~positive regulation of developmental process	6	0.049009621	MEN1, WNT7B, CD36, GATA6, FOXP3, NKX2-5	2.999779233	1	0.646024492	55.03464335
GO:0045121~membrane raft	4	0.049267886	CAV3, ADCY3, CD36, SGCA	4.823143684	0.999952416	0.669084139	46.83050674

F. Enriched GO terms of down-regulated DEGs in short-term PFC.

Term	Count	PValue	Genes	Fold Enrichment	Bonferroni	Benjamini	FDR
GO:0003700~transcription factor activity	21	0.001039075	ZBTB7B, ISX, E2F2, GSX2, NKX2-6, MTA3, GABPA, CBFA2T3, HOXD11, MSX2, IRF9, HAND1, ZSCAN21, ZGLP1, HOPX, CREB3L2, LHX6, MESP2, LBX2, LHX9, NFATC1	2.233527566	0.279261426	0.279261426	1.389823895
GO:0030528~transcription regulator activity	25	0.009717098	ISX, E2F2, CBFA2T3, HOXD11, MSX2, HAND1, ZSCAN21, CREB3L2, LHX6, LHX9, NFATC1, ZBTB7B, UTF1, GSX2, NKX2-6, MTA3, GABPA, PRPF6, IRF9, BHLHA9, ZGLP1, HOPX, MAP3K10, LBX2, MESP2	1.710907162	0.953849813	0.785174053	12.31814871
GO:0043565~sequence-specific DNA binding	14	0.016880474	ISX, GSX2, NKX2-6, MTA3, GABPA, HOXD11, MSX2, ZGLP1, HOPX, CREB3L2, LHX6, LHX9, LBX2, NFATC1	2.078198311	0.995311959	0.832635328	20.48239488
GO:0048663~neuron fate commitment	4	0.018687853	WNT1, GSX2, LHX6, DLL1	7.051375195	1	1	26.24383581
GO:0045449~regulation of transcription	38	0.021818966	ISX, E2F2, ZBTB33, CBFA2T3, LIN28A, HOXD11, MSX2, HAND1, ZSCAN21,	1.413757981	1	0.999997823	29.95095957

			CREB3L2, CASZ1, LHX6, LHX9, NFATC1, ZBTB7B, KAT2A, IRAK1, MAGEL2, UTF1, IL5, GSX2, NKX2-6, MTA3, GABPA, KLF14, MBD1, PRPF6, IRF9, BHLHA9, ZGLP1, SP4, MAP3K10, HOPX, BRDT, MESP2, LBX2, KDM4D, SUDS3				
GO:0043009~chordate embryonic development	11	0.030510553	KAT2A, HBA-A2, WNT1, HAND1, NKX2-6, VANGL2, GABPA, HOPX, DLL1, AMBRA1, MESP2	2.164822432	1	0.999995011	39.34654581
GO:0051252~regulation of RNA metabolic process	27	0.031530759	ISX, E2F2, RBM5, LIN28A, CBFA2T3, HOXD11, MSX2, HAND1, ZSCAN21, CREB3L2, LHX6, LHX9, NFATC1, ZBTB7B, KAT2A, UTF1, IL5, GSX2, NKX2-6, MTA3, GABPA, PRPF6, IRF9, ZGLP1, HOPX, LBX2, MESP2	1.503392998	1	0.999922667	40.36830412
GO:0045165~cell fate commitment	6	0.032053724	WNT1, HAND1, GSX2, LHX6, DLL1, HOXD11	3.381781981	1	0.999547898	40.88578367
GO:0009792~embryonic development ending in birth or egg	11	0.032249054	KAT2A, HBA-A2, WNT1, HAND1, NKX2-6, VANGL2, GABPA, HOPX, DLL1, AMBRA1,	2.144447633	1	0.998431696	41.07798188

hatching			MESP2				
GO:0008380~RNA splicing	7	0.034235454	PPIH, RBM5, CELF2, WBP11, LSM10, CWC22, PRPF6	2.885450795	1	0.997211287	42.99952685
GO:0045095~keratin filament	4	0.038747262	KRT81, GM11938, KRT86, KRTAP10-10	5.316326531	0.999247613	0.999247613	38.59379534
GO:0006355~regulation of transcription, DNA-dependent	26	0.044217415	ISX, E2F2, LIN28A, CBFA2T3, HOXD11, MSX2, HAND1, ZSCAN21, CREB3L2, LHX6, LHX9, NFATC1, ZBTB7B, KAT2A, UTF1, IL5, GSX2, NKX2-6, MTA3, GABPA, PRPF6, IRF9, ZGLP1, HOPX, LBX2, MESP2	1.470440356	1	0.998746689	51.79753428
GO:0003002~regionalization	7	0.044246595	KAT2A, WNT1, GSX2, DLL1, SP8, MESP2, HOXD11	2.710166401	1	0.997377259	51.82127534
GO:0006879~cellular iron ion homeostasis	3	0.047000551	1600014K23RIK, ALAS2, HAMP	8.571068124	1	0.996621288	54.01319355
GO:0005179~hormone activity	5	0.047565088	SCT, NTS, HAMP, IGF2, INSL6	3.651954048	0.999999785	0.978458279	48.11136188

G. Enriched GO terms of up-regulated DEGs in short-term hippocampus.

Term	Count	PValue	Genes	Fold Enrichment	Bonferroni	Benjamini	FDR
GO:0044265~cellular macromolecule catabolic process	9	0.010762443	SENP3, RNF6, USPL1, ZCCHC11, USP5, ADAM17, CDC23, DNASE1L2, USP32	2.910259156	0.999038426	0.999038426	14.89582135
GO:0009057~macromolecule catabolic process	9	0.015996502	SENP3, RNF6, USPL1, ZCCHC11, USP5, ADAM17, CDC23, DNASE1L2, USP32	2.710011966	0.999968096	0.994351643	21.36638577
GO:0030054~cell junction	7	0.019632138	SYNPR, OCLN, GABRA3, GABRB2, MPP7, HOMER2, GAK	3.210858401	0.945775854	0.945775854	21.0175443
GO:0008270~zinc ion binding	18	0.025609357	ZFP62, BRD1, ZCCHC11, CAR13, USP5, ZHX1, ZFP719, TRIM23, ZIC1, DMRTB1, ZIC3, ZIC2, RNF6, ZSCAN21, ZFP191, NBR1, ADAM17, ADAM9	1.695919453	0.990865348	0.990865348	27.37405138
GO:0005615~extracellular space	7	0.028203017	BMP4, CCL3, CXCL5, PSAP, GPX3, ADAM9, SPP1	2.953235711	0.985085616	0.877875539	28.85406716
GO:0043167~ion binding	28	0.0325581	ZFP62, MMGT1, GABRB2, USP5, ZFP719, ZIC1, CANX, ZIC3, CALU, ZIC2, P4HA1, ZSCAN21, ZFP191, ACSL3, PLS3, ADAM9, BRD1,	1.411589738	0.997499047	0.949990467	33.50774722

			ZCCHC11, CAR13, GABRA3, ZHX1, CACNG5, TRIM23, DMRTB1, RNF6, NBR1, ADAM17, NEK6				
GO:0007049~cell cycle	8	0.032564621	CDKN1C, TXNIP, CAMK2D, AHCTF1, CDC23, CABLES1, NEK6, GAK	2.57842928	0.999999999	0.999162277	38.9505706
GO:0006511~ubiquitin-dependent protein catabolic process	4	0.033602014	RNF6, USPL1, USP5, USP32	5.586596773	1	0.995854847	39.91913309
GO:0000278~mitotic cell cycle	5	0.033793392	CAMK2D, AHCTF1, CDC23, CABLES1, NEK6	4.035400333	1	0.987894342	40.09623969
GO:0019887~protein kinase regulator activity	3	0.034672358	CDKN1C, CABLES1, TRIB2	10.08449279	0.998316829	0.881046803	35.27728056
GO:0005783~endoplasmic reticulum	9	0.03519508	MMGT1, SLN, P4HA1, A130022J15RIK, HSPA5, ACSL3, CANX, DERL3, CALU	2.315364991	0.994840496	0.827203089	34.71227836
GO:0005576~extracellular region	14	0.036354249	BMP4, CCL3, IGFBPL1, CXCL5, PSAP, DNASE1L2, CALU, SOST, A130022J15RIK, GPX3, ADAM17, HSPA5, SPP1, ADAM9	1.796551724	0.995676262	0.743572355	35.63962282
GO:0005788~endoplasmic reticulum lumen	3	0.042583362	P4HA1, HSPA5, CALU	8.982758621	0.998333357	0.721792694	40.41963146

GO:0022402~cell cycle process	6	0.046664982	CDKN1C, CAMK2D, AHCTF1, CDC23, CABLES1, NEK6	3.006527271	1	0.993984477	50.95045713
GO:0004221~ubiquitin thiolesterase activity	3	0.048492156	USPL1, USP5, USP32	8.380071474	0.999876223	0.894522488	45.81878183

H. Enriched GO terms of down-regulated DEGs in short-term hippocampus.

Term	Count	PValue	Genes	Fold Enrichment	Bonferroni	Benjamini	FDR
GO:0000785~chromatin	4	0.003291125	SIRT6, 1700014N06RIK, HMGA1, CHD3	12.63030303	0.160305618	0.160305618	3.165108891
GO:0006333~chromatin assembly or disassembly	3	0.008016087	SET, 1700014N06RIK, CHD3	20.77675841	0.761315608	0.761315608	9.421280635
GO:0044427~chromosomal part	4	0.019798113	SIRT6, 1700014N06RIK, HMGA1, CHD3	6.553459119	0.653483991	0.411343896	17.72459723
GO:0019941~modification-dependent protein catabolic process	4	0.023899893	DZIP3, GM2933, PCNP, FBXL13	5.944006999	0.986510491	0.883855654	25.72572072
GO:0043632~modification-dependent macromolecule catabolic process	4	0.023899893	DZIP3, GM2933, PCNP, FBXL13	5.944006999	0.986510491	0.883855654	25.72572072
GO:0051603~proteolysis involved in cellular protein catabolic process	4	0.027215179	DZIP3, GM2933, PCNP, FBXL13	5.654598419	0.992638136	0.805465817	28.76844854
GO:0044257~cellular protein catabolic process	4	0.027613104	DZIP3, GM2933, PCNP, FBXL13	5.623008483	0.993155222	0.712366249	29.12585646
GO:0030163~protein catabolic process	4	0.03020727	DZIP3, GM2933, PCNP, FBXL13	5.430855316	0.9957455	0.664440373	31.41580029
GO:0005694~chromosome	4	0.03100523	SIRT6, 1700014N06RIK, HMGA1, CHD3	5.513227513	0.811619498	0.426748346	26.45636772

GO:0044265~cellular macromolecule catabolic process	4	0.038117579	DZIP3, GM2933, PCNP, FBXL13	4.958219303	0.999009821	0.684291961	37.98548773
GO:0009057~macromolecule catabolic process	4	0.045606711	DZIP3, GM2933, PCNP, FBXL13	4.617057424	0.999753695	0.694862102	43.66759462
